# Masu salmon species complex relationships and sex chromosomes revealed from analyses of the masu salmon (*Oncorhynchus masou masou*) genome assembly

**DOI:** 10.1093/g3journal/jkae278

**Published:** 2024-11-28

**Authors:** Kris A Christensen, Anne-Marie Flores, Jay Joshi, Kiko Shibata, Takafumi Fujimoto, Ben F Koop, Robert H Devlin

**Affiliations:** Department of Biology, University of Victoria, Victoria, British Columbia, Canada V8W 2Y2; Department of Biology, University of Victoria, Victoria, British Columbia, Canada V8W 2Y2; Department of Biology, University of Victoria, Victoria, British Columbia, Canada V8W 2Y2; Faculty and Graduate School of Fisheries Sciences, Hokkaido University, Hakodate, Hokkaido 041-8611, Japan; Faculty and Graduate School of Fisheries Sciences, Hokkaido University, Hakodate, Hokkaido 041-8611, Japan; Department of Biology, University of Victoria, Victoria, British Columbia, Canada V8W 2Y2; Fisheries and Oceans Canada, West Vancouver, British Columbia, Canada V7V 1N6

**Keywords:** cherry salmon, *Oncorhynchus*, masu salmon, species complex, salmonid evolution, Pacific salmon

## Abstract

Masu salmon (*Oncorhynchus masou*) are the only Pacific salmon endemic to Asia. Some researchers prefer to categorize these salmon into 4 subspecies (masu—*Oncorhynchus masou masou*, amago—*Oncorhynchus masou ishikawae*, Biwa—*Oncorhynchus masou* subsp., and Formosan—*Oncorhynchus masou formosanus*), while others prefer individual species designations. Even though the masu salmon fishery is thousands of years old, classification of the diversity within the masu salmon species complex remains elusive. In this study, a genetic map and reference genome assembly were generated for 1 species/subspecies (masu) to provide resources for understanding the species complex. In *O. m. masou*, the sex chromosome was determined to be chromosome 7. Resequenced genomes from 2 other putative subspecies (amago and Biwa) provided evidence that they do not share the same sex chromosome. Principal component and admixture analyses clustered the amago and Biwa salmon close together. This supported previous findings of a close relationship between amago and Biwa salmon and a more distant relationship to masu salmon for both. Additional analyses of the masu salmon species complex will benefit from using the new reference genome assembly.

## Introduction

Interest in masu salmon (*Oncorhynchus masou*) has often been related to fisheries (Miyakoshi *et al*. 2001; Mizuno *et al*. 2004), aquaculture (Choe and Yamazaki 1998), conservation (Kottelat 1996; Ando *et al*. 2002; Chung *et al*. 2008; Nagata *et al*. 2012; Ho *et al*. 2016; Kato-Unoki *et al*. 2020; Liang *et al*. 2023), and its unique and complex evolutionary history (McKay *et al*. 1996; Oohara *et al*. 1997; Kitanishi *et al*. 2007). In Japan, there is evidence that people subsisted on salmon, including masu salmon, for 5,500 years (Nagata *et al*. 2012). Masu salmon are commonly thought to be a species complex (Kimura 1990; Kato 1991; Nakabo 2009), and the complex has a range that includes parts of Russia, China, Japan, Taiwan, North Korea, and South Korea (Kato 1991; Gwo *et al*. 2008; Nakabo 2009). Members of this complex are the only Pacific salmon endemic to Asia (Gwo *et al*. 2008; Malyutina *et al*. 2009; Kawanabe *et al*. 2012; Tabata *et al*. 2016).

Masu salmon diverged from the other Pacific salmon roughly 10 million years ago, possibly before the divergence of other modern salmonid species (McKay *et al*. 1996; Oohara *et al*. 1997; Osinov and Lebedev 2000; Wilson and Turner 2009). Four subspecies of masu salmon have been proposed (masu—*Oncorhynchus masou masou*, Biwa—*Oncorhynchus masou* subsp., amago—*Oncorhynchus masou*  *ishikawae*, and Formosan—*Oncorhynchus masou*  *formosanus*) based on distinct morphology and ranges (Kato 1991; Nakabo 2009). Recent data supported only weak genetic structure among some subspecies and a recent range expansion in the last 100,000–150,000 years (Ho *et al*. 2016; Gong *et al*. 2017; Yamamoto *et al*. 2020). Only masu salmon from Lake Biwa, the largest and oldest Japanese lake (Kawanabe *et al*. 2012), had a unique mtDNA haplotype (Kuwahara *et al*. 2019; Yamamoto *et al*. 2020). Based on microsatellite evidence, the Lake Biwa and amago subspecies, and the masu and Formosan subspecies are each more closely related to each other than to the other subspecies (Yamamoto *et al*. 2020).

Debate about the classification of the masu salmon complex as either subspecies or species is ongoing (e.g. Gwo *et al*. 2008; Nakabo 2009; Kuwahara *et al*. 2019). While characterizing a species complex into distinct subspecies or species may not be possible, government regulation may benefit from these designations. In the United States and Canada, there are frameworks for conservation of populations below the species level (reviewed in Winker *et al*. 2007), but support for conservation may diminish if the species is not well defined (reviewed in George and Mayden 2005). Understanding the genetic and phenotypic variation in the species complex is significant for determining the proper criteria for categorization, if categorization is possible.

In addition to variation among subspecies, there is variation in migration life history (e.g. sea-run and landlocked) (Kato 1991). In some publications, the landlocked salmon is masu (masu translates to trout in Japanese), and the sea-run salmon is the cherry salmon, based on the distinctive red coloration during spawning (Usui *et al*. 2022). This division between landlocked and sea-run masu salmon is similar to that between rainbow trout (*Oncorhynchus*  *mykiss*) and steelhead or between kokanee (*Oncorhynchus nerka*) and sockeye salmon. The different migratory ecotypes of masu salmon, landlocked and sea-run, could be influenced by environmental conditions (Ugachi *et al*. 2023) that vary with latitude (Kitanishi *et al*. 2007; Malyutina *et al*. 2009; Morita *et al*. 2014). Other life history variations can include precocious parr and variation in the ages of migration (Kato 1991).

The goal of this study was to provide resources to understand the nature of the relationships among proposed masu salmon subspecies more fully. To this end, we generated a reference genome assembly for *O. m. masou* from Nanopore and Hi-C data (NCBI: GCF_036934945.1). The masu salmon was the last Pacific salmon species without a chromosome-level reference genome assembly, and therefore with the completion of this work, all Pacific salmon species now have publicly available, chromosome-level reference genome assemblies (i.e. Chinook (Christensen *et al*. 2018), coho (Rondeau *et al*. 2023b), chum (Rondeau *et al*. 2023a), pink (Christensen *et al*. 2021), sockeye (Christensen *et al*. 2020), and masu salmon). Genome assemblies provide several advantages over using traditional genetic tools, including understanding the genomic context of a genetic marker (e.g. genic or intergenic), targeted marker development, and high-throughput marker development.

## Methods

### Genome assemblies

Sampling of masu salmon at the Nanae Freshwater Station, Hokkaido University, was performed according to the Guide for the Care and Use of Laboratory Animals in Hokkaido University (approval number 29-3). Salmon sampled for tissue in this work were reared in standard aquaculture conditions. Salmon were netted from rearing tanks and rapidly euthanized by a blow to the cranium.

Masu salmon (*O. m. masou*) broodstock of the Nanae Freshwater Station at Hokkaido University (fertilized in 2017) were collected and euthanized on October 9, 2020. Four males and 4 females were sampled. Heart, spleen, muscle, kidney, intestine, gill, brain, liver, and gonad tissues were collected during dissection. Amago (*O. m. ishikawae*) and Biwa salmon (*O. m.* subsp.) were purchased frozen from the Shiga Prefecture Samegai Trout Farm on October 7, 2020. There were 5 males and 3 females of amago salmon, and 4 males and 4 females of Biwa salmon. Tissues were immediately frozen and stored at −80°C.

High molecular weight DNA was extracted from multiple salmon samples using the Circulomics animal tissue kits (now PacBio Nanobind tissue kits). This ensured that high-quality DNA could be collected and that there would be enough DNA for sequencing. Libraries were produced from the extracted DNA using the Oxford Nanopore Technology (ONT) Ligation Sequencing Kits (SQK-LSK109 and SQK-LSK110). The libraries were sequenced with a flowcell (FLO-MIN106) on a MinION (ONT).

A Hi-C library was produced from the muscle tissue of a male *O. m. masou* and sequenced at the Michael Smith Genome Sciences Centre (available on the NCBI: SRX23885553). The Hi-C library was produced using the Arima Hi-C 2.0 kit, the Swift Biosciences Accel-NGS S2S Plus DNA Library Kit, and an indexing kit. The library was amplified with the NEBNext Q5 Mastermix (supplemented with 2 mM MgSO_4_). Sequencing was performed on a NovaSeq 6000 (PE150).

Whole genome shotgun sequencing libraries were prepared and sequenced at the Centre d’expertise et de services Génome Québec using the HMW gDNA extractions for 3 salmon, 1 for each subspecies. The libraries were sequenced on a NovaSeq (PE150). The short paired-end reads were quality trimmed and filtered using Trimmomatic (version 0.38) (Bolger *et al*. 2014). The following parameters were used: ILLUMINACLIP (TruSeq3-PE) 2:30:10:2:keepBothReads, leading=3, trailing=3, minlen=36.

An initial genome assembly was produced using Flye (version 2.8.2) (Kolmogorov *et al*. 2019) from ONT reads from multiple individuals (as DNA was limited per individual). The following parameters were used: asm-coverage=30, nano-raw, and g=2.4g. The initial assembly was then polished once using racon (version 1.4.16, default settings) (Vaser *et al*. 2017) with the raw ONT reads aligned to the assembly using minimap2 (parameters: x=map-ont) (Li 2018). The assembly was then polished twice using Pilon (version 1.22, default settings) (Walker *et al*. 2014) with the Illumina short paired-end reads (see above) that were aligned to the assembly with bwa mem (version 0.7.17, -M parameter) (Li and Durbin 2009; Li 2013) and sorted and indexed with Samtools (version 1.12) (Li *et al*. 2009).

We used Salsa (parameters: e=GATCGATC,GANTGATC,GANTANTC,GATCANTC) (Ghurye *et al*. 2017, 2019) to scaffold the contigs using Hi-C data (see above). A genetic map (see below) was then used to order and orient contigs/scaffolds using Chromonomer (version 1.10) (Catchen *et al*. 2020). A contact map of the Hi-C data was used to refine the order and orientation of scaffolds produced by Salsa and the genetic map. The contact map was manually reviewed in Juicebox (version 1.11.08) (Durand *et al*. 2016). To produce the Hi-C contact map, we used the Arima Genomics (github.com/ArimaGenomics/mapping_pipeline 2021) pipeline to map Hi-C reads to the genome assembly. Phase Genomics (github.com/phasegenomics/juicebox_scripts 2021) scripts were used to produce the contact map and to convert the output from Juicebox into the AGP and FASTA files submitted to the NCBI (GCF_036934945.1; Sayers *et al*. 2022). During the submission process, the NCBI identifies adapter sequences and potential contamination. If any of these were identified, they were removed. The NCBI also provided annotation of the genome assembly using version 10.2 of the NCBI Eukaryotic Genome Annotation Pipeline (https://www.ncbi.nlm.nih.gov/refseq/annotation_euk/Oncorhynchus_masou_masou/GCF_036934945.1-RS_2024_04/).

Haplotig and artifact contigs were identified in the genome assembly with the purge_haplotigs pipeline (Roach *et al*. 2018). This was performed after submission of the genome assembly to the NCBI based on a reviewer's recommendation. Briefly, minimap2 (parameters: ax=sr) (Li 2018) was used to align paired-end reads (used for polishing) to the genome assembly. The purge_haplotig pipeline (parameters: l=10, m=28, h=85) was then used to identify the haplotigs or artifacts based on coverage and sequence similarity.

Expected genome sizes were either taken from the animal genome size database (genomesize.com) or from estimating the genome size using a kmer based approach. The kmer estimation was performed using the R package findGSEP (version 1.2.0, parameters: sizek=21, ploidy=2, exp_hom=41, xlimit=-1, ylimit=-1) (Sun *et al*. 2018) after a kmer histogram was produced using Jellyfish (version 2.2.4, parameters: count C, m=21, s=1000000; histo h=2000000) (Marçais and Kingsford 2011).

### Genetic maps

In September 2021, 4 single-cross families were generated with the Mori strain of Masu salmon (*O. m. masou*) at the Nanae Freshwater Station. Parents and progeny were euthanized and tissues were stored in ethanol (November 19, 2021). DNA was extracted from families using the NucleoSpin Tissue Mini kit (Macherey-Nagel).

Whole genome sequencing was performed at the Beijing Genomics Institute (BGI) for each family (including parents) and 5 other masu (*O. m. masou*), Biwa (*O. m.* subsp.), and amago salmon (*O. m. ishikawae*) that had not already been sequenced using short read technology (see ‘*Genome assemblies*’). Three males and 3 females per subspecies were sequenced (excluding the masu salmon families). Paired-end libraries were prepared and sequenced at BGI (DNBseq, PE150). Reads were also filtered at BGI by removing low-quality reads, adapter sequences, and potential contamination using SOAPnuke (parameters: n=0.001, l=10, q=0.4, adaMR=0.25, and ada_trim) (Chen *et al*. 2018). The final number of individuals with resequenced genomes included individuals from 4 families and 3 males and females of each species/subspecies (n = 168, Supplementary Table 1). The progeny of the families was not sexed.

DNBseq and Illumina reads from each salmon (see ‘*Genome assemblies*’ section) were aligned to a version of the genome assembly after scaffolding with Salsa (see ‘*Genome assemblies*’). The genetic map was used to scaffold this version of the assembly after the genetic map was complete. The final version of the genome assembly was not available until the genetic map was completed. The alignment files were produced using bwa mem (parameter: -M) and sorted using Samtools (version 1.12). Picard (version 2.26.3, parameter: lenient validation stringency) (Broad Institute 2019) was applied to add read group information and flag potential PCR duplicates. GATK (version 3.8) (McKenna *et al*. 2010; DePristo *et al*. 2011; Van der Auwera *et al*. 2013) was used to call nucleotide variants. The HaplotypeCaller module in GATK (parameters: genotyping_mode=DISCOVERY, emitRefConfidence=GVCF) was called for each sample, and the GenotypeGVCF module (default parameters) was used to genotype the combined samples. Variants were filtered using a stringent filter for the initial genetic map using VCFtools (version 0.1.16, parameters: min-alleles=2, max-alleles=2, max-missing=0.9, maf=0.25, max-maf=0.75, min-meanDP=8, max-meanDP=30) (Danecek *et al*. 2011). Lower stringency filters were also attempted (e.g. a minor allele frequency of 0.05 was used to confirm expected relatedness using VCFtools). Homologous regions negatively impact mapping quality scores and SNP calling in these regions (Christensen *et al*. 2024). This appears to be confined to small regions relative to the genome (Christensen *et al*. 2024). It is unclear how this might impact genetic mapping or if using longer reads could reduce the impact.

To generate the initial genetic map, we first called parental genotypes and error corrected the genotypes using the ParentCall2 module of Lep-MAP3 (Rastas 2017) (parameters: removeNonInformative=1). Markers were then filtered using the Filtering2 module (parameters: dataTolerance=0.001, removeNonInformative=1). Finally, we clustered markers into linkage groups using the SeparateChromosomes2 module (parameters: sizeLimit=50, lodLimit=10). After converting the data to the proper format, we utilized Chromonomer (parameter: disable_splitting) to order contigs based on the genetic map. This genetic map was fragmented and did not have the expected number of linkage group numbers, reducing its value for placing contigs in the genome assembly. This was possibly due to inbreeding in the families used to generate the genetic maps (see ‘*Results and discussion*’). We also tried to generate sex-specific genetic maps from individual families but the maps were still fragmented.

SNP calling was performed again with the final genome assembly (submitted to the NCBI) to produce a genome-wide genetic map. Filtering of these SNPs used the following parameters in VCFtools: max-alleles=2, min-alleles=2, max-missing=0.9, min-meanDP=5, max-meanDP=100, and maf=0.01. Female and male genetic maps were produced using the order of the genetic markers on the genome assembly. Recombination events of these maps were plotted using ggplot2 (Wickham 2016), reshape2 (Wickham 2007), and gridExtra (Auguie 2017) packages in R (R Core Team 2024).

### Other analyses

To identify putative sex chromosomes (allosomes), *F*_st_ was estimated between male and female masu salmon with VCFtools in windows along the genome (parameters: weir-fst-pop, fst-window-size=500000, fst-window-step=250000). The results were visualized using the qqman package (Turner 2018) in R. Thinned genotypes (allowing only 1 SNP within a specified distance from each other) of the SNPs used for the genetic map (VCFtools parameters: thin=1000) were visualized using IGV (Thorvaldsdóttir *et al*. 2013). Thinning was done to allow a larger section to be visualized with existing hardware. The genotypes from the largest *F*_st_ peak were compared to the unplaced scaffold with the sdY gene—the sex-determining gene in salmonids (Yano *et al*. 2013) (Hi-C data was unable to place the scaffold with sdY). The location of the sdY gene was identified by aligning the sdY protein (NP_001268345.1—rainbow trout sdY) to the genome assembly using TBLASTN. Identifying the putative sex chromosome was repeated with Biwa and amago salmon.

Admixture and principal component analyses (PCA) were performed on the SNPs used for the final genetic map (see previous section) after filtering for linkage disequilibrium (LD). SNPs were filtered for LD, as markers need to be independent for admixture analysis, and PCA can be influenced by SNPs in large LD blocks (e.g. inversions). However, all markers were used for comparison to the LD-filtered dataset for PCA, and we also compared a PCA with and without the Masu salmon families. To filter for LD, BCFtools (Danecek *et al*. 2021) was used with the +prune add-on (parameters: w=20kb, n=2, and l=0.4). There were 176,118 SNPs after this filter.

For the admixture analysis, PLINK (version 1.9, parameters: double-id, and allow-extra-chr) (Chang *et al*. 2015) and bash commands were used to convert the SNPs to the appropriate format. Admixture (version 1.3, parameters: cv) (Alexander *et al*. 2009; Zhou *et al*. 2011) was tested with 1–20 putative clusters. The lowest cross-validation score was accepted as the criteria for choosing the best-supported cluster value (k = 5). The ggplot2 package was used to visualize the admixture results. PLINK was used for the PCA analysis, and the ggplot2 and ggrepel (Slowikowski 2021) packages were used to visualize.

Information about the SNP coverage (the number of reads used to call a variant) and counts of the genotypes per individual were calculated from the filtered variants using a custom Python script (github.com/KrisChristensen/VCFstats). The ggplot2 R package was used to visualize the average percent of alternative homozygous genotype for each putative subspecies. Finally, runs of homozygosity (ROH) were identified using PLINK (parameters: homozyg-snp=50, homozyg-kb=500). The estimation of the percent of the genome in ROH was calculated as the sum of ROH per individual divided by an estimated genome size of 2.7 Gb and multiplied by 100.

A comparison of chromosomal fusions and fissions was performed by aligning salmonid genome assemblies together using Dgenies (Cabanettes and Klopp 2018). The following genome assemblies were utilized: rainbow trout (GCF_013265735.2 (Gao *et al*. 2021)), Chinook salmon—*Oncorhynchus tshawytscha* (GCF_018296145.1 (Christensen *et al*. 2018)), coho salmon—*Oncorhynchus kisutch* (GCF_002021735.2 (Rondeau *et al*. 2023b)), pink salmon (GCF_021184085.1 (Christensen *et al*. 2021)), chum salmon (GCF_023373465.1 (Rondeau *et al*. 2023a)), and sockeye salmon (GCF_006149115.2 (Christensen *et al*. 2020)). The northern pike (*Esox lucius*) genome assembly (GCF_011004845.1—vertebrategenomesproject.org) was employed as a proxy for an ancestral genome in this analysis (Campbell *et al*. 2013; Rondeau *et al*. 2014).

## Results and discussion

With the completion of the masu salmon genome assembly, all of the Pacific salmon now have a reference genome assembly publicly available (Christensen *et al*. 2018, 2020, 2021; Rondeau *et al*. 2023b, 2023a). The availability of these annotated genomes facilitates the transfer of knowledge among species (e.g. the sequence of a gene of interest will be known for all species in most instances). In addition, the masu salmon genome assembly is a resource that will be useful for research among proposed subspecies of masu salmon to improve our understanding of their unique and complex evolutionary history.

### Insights from the masu (*O. m. masou*) genome assembly

The diploid ancestor of salmonids is thought to have had 24 chromosome pairs (reviewed in Phillips and Ráb 2001), similar to the 25 pairs in northern pike (Rondeau *et al*. 2014). After an ancestral salmonid whole genome duplication (Allendorf and Thorgaard 1984), there would have been 48 pairs. Modern Pacific salmon have 26–37 chromosome pairs due to chromosomal fusion and fission events after the ancestral genome duplication. The number of chromosome pairs can vary within a species as well (e.g. Thorgaard 1978, 1983).

The estimated genome sizes of masu salmon ranged from 2.0–3.2 Gb based on C-values (Ojima *et al*. 1963; Johnson *et al*. 1987; Ojima and Yamamoto 1990), with an average of 2.7 Gb. The estimated genome size of our sample based on a kmer analysis was 2.5 Gb. The *O. m. masou* genome assembly is 2.7 Gb long and comprised of 33 chromosomes (Hi-C contact map available in Supplementary Fig. 1), with a contig N50 of 2.5 Mb, a scaffold N50 of 66.3 Mb, and 98.9% complete BUSCOs (Table 1). There were 59.1% single copy and 39.8% duplicated complete BUSCOs, and only 0.5% fragmented and 0.6% missing. We identified 2,709 haplotig (∼32 Mb) and 2,196 artifact contigs (∼32 Mb), which represent ∼2.4% of sequence data (Supplementary File 1). The metrics of this assembly are better than an *O. m. ishikawae* genome assembly at the scaffold stage that was also available on the NCBI (GCA_036245685.1—Noda *et al*. 2024). The length of the *O. m. ishikawae* assembly is 2.4 Gb, with a contig N50 of 128.1 kb and a scaffold N50 of 47.5 Mb.

**Table 1.** Genome assembly metrics.

Subspecies	Metric	Value
Masu (*O. m. masou*)—reference	Total length (Gb)^a^	2.7
	Contig N50 (Mb)^a^	2.5
	Contigs^a^	21,300
	Complete BUSCO (4.1.4)^a^	0.99

^a^Values taken from NCBI (GCF_036934945.1).

During annotation of the genome assembly produced in this study, the NCBI identified 40,543 protein-coding genes, which is consistent with other Pacific salmon and the rainbow trout genome assembly. The number of protein-coding genes in Pacific salmon genome assemblies ranged from 39,464–41,269 (GCF_018296145.1, GCF_023373465.1, GCF_034236695.1, GCF_002021735.2, GCF_021184085.1). The reference rainbow trout genome assembly had 41,896 annotated protein-coding genes (GCF_013265735.2).

**Fig. 1.**
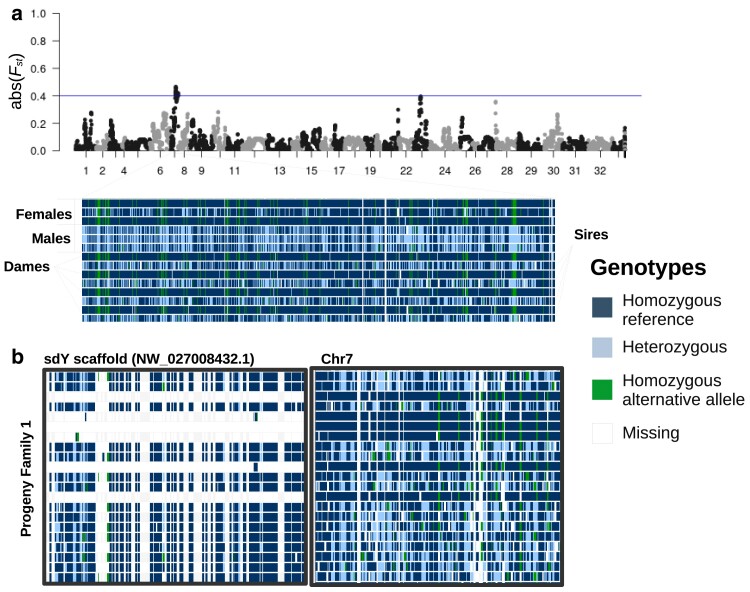
Sex chromosome of masu salmon (*O. m. masou*). a) Manhattan plot of windowed *F*_st_ values (window 500 kb, step 250 kb) between 7 male and 7 female salmon. The *F*_st_ values were shown as the absolute values so all values could be plotted. The highest peak was at the end of chromosome 7. The horizontal line represents an *F*_st_ value of 0.4. Below the Manhattan plot is a screenshot with genotypes plotted in IGV. Females have an excess of homozygous genotypes and males have an excess of heterozygous genotypes in the region where the largest *F*_st_ peak is on chromosome 7. The individuals labeled sires and dams are the parents of the crosses used for the genetic maps. b) A screenshot of the genotypes of some progeny from family 1. These individuals were not sexed, as they were too young, but unfiltered genotypes reveal the presence or absence of the sdY gene. The presence or absence of sdY matches haplotypes from chromosome 7.

The sex-determining gene in salmonids (sdY) (Yano *et al*. 2013) was not placed onto a chromosome in the genome assembly, and the sex chromosomes were unknown before the present study. To identify the sex chromosomes of the *O. m. masou* genome assembly (collapsed as this was a haploid assembly), we used several complementary analyses. In 1 evaluation, we examined genetic differentiation between males and females. Identifying that *F*_st_ was greatest between male and female masu salmon at the end of chromosome 7 (Fig. 1a). A second approach was to identify males and females based on their genotypes near the sdY gene and then compare these genotypes with putative sex chromosomes. Males and females are expected to have different genotype patterns near the sex-determining region, with males being more heterozygous for mutations that accumulate on the Y-chromosome. Genotypes were linked between the sdY unplaced scaffold (NW_027008432.1) and chromosome 7 (Fig. 1b). Also, there were no male recombination events detected at this end of chromosome 7 in the genetic map (Supplementary Fig. 2). These analyses support that chromosome 7 is the masu salmon sex chromosome (collapsed).

**Fig. 2.**
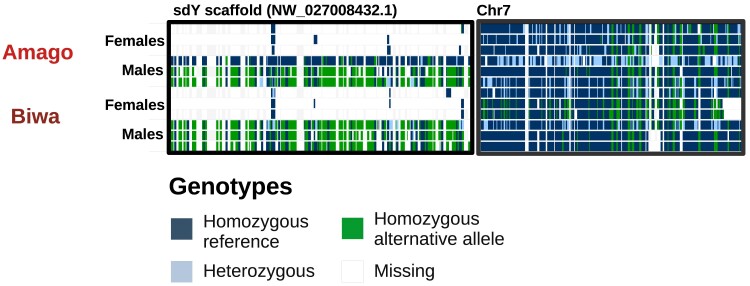
Evidence of alternative sex chromosome(s). IGV screenshots of genotypes of amago and Biwa salmon. Unlike masu salmon, these 2 putative subspecies do not have linked genotypes between the sdY unplaced scaffold and chromosome 7.

**Fig. 3.**
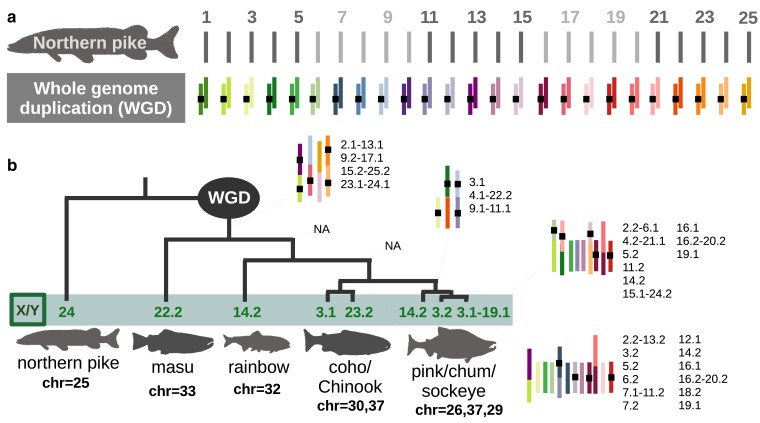
Comparison of syntenic chromosomes in Pacific salmon. a) In this comparison, all the reference genome assemblies for each species were aligned to the northern pike reference assembly. This was done so chromosomal rearrangements could be directly compared among species using a common nomenclature. Each chromosome from every species is depicted and named based on its synteny with the northern pike. We used the designation of 1 (with box) and 2 to depict the first and second homolog (since for every northern pike chromosome, salmon have two due to an ancestral whole genome duplication). These assignments were taken from Sutherland *et al*. (2016). b) A simplified and unscaled phylogeny adapted from several studies (Stearley and Smith 1993; Wilson and Turner 2009; Crête-Lafrenière et al. 2012; Sutherland *et al*. 2016) showing chromosomes without changes in fusions or fissions for different branches of the phylogeny (i.e. shared interspecific chromosomal rearrangements). The sex chromosomes (located below the phylogeny) were determined from multiple studies in addition to the current (Phillips *et al*. 2005, 2006; Rexroad *et al*. 2008; Naish *et al*. 2013; Brieuc *et al*. 2014; Kodama *et al*. 2014; Palti *et al*. 2015; Larson *et al*. 2016; Sutherland *et al*. 2017; Pan *et al*. 2019; Christensen *et al*. 2021; Gao *et al*. 2021; Rondeau *et al*. 2023a). The number of chromosomes for each genome assembly is specified below their name.

Notably, the sdY and chromosome 7 genotypes were not linked in amago or Biwa salmon (Fig. 2). There were not enough samples from these 2 subspecies to unambiguously identify their sex chromosome(s), but the disagreement of genotypes between sdY and chromosome 7 suggests an alternative sex chromosome. Masu salmon do not share a sex chromosome with other species or subspecies examined in this study (Fig. 3)

From previous work (Phillips *et al*. 2005; Davidson *et al*. 2009; Phillips 2013; Yano *et al*. 2013; Eisbrenner *et al*. 2014; Sutherland *et al*. 2016, 2017), it was well understood that transpositions of the sex-determining region were common in salmonid evolutionary history. We identified 2 potential transpositions in the masu salmon complex. The sdY gene was localized to chromosome 7 in the *O. m. masou* subspecies (supported by 3 different analyses). In the other 2 subspecies, the location was unknown but likely different from the *O. m. masou*.

Researchers believe the sdY gene jumps to different autosomes as a transposable cassette (reviewed in Bertho *et al*. 2021). They have postulated that a jumping sex locus could prevent sex chromosome degeneration (Bertho *et al*. 2021). Sexually antagonistic selection may help to explain the spread of a new sex-determining locus (van Doorn and Kirkpatrick 2007). For example, if a linked allele on the novel Y-chromosome confers a fitness advantage to males, the advantage may drive the spread of the novel Y-chromosome. While transpositions among populations of Atlantic salmon (*Salmo salar*) have previously been noted (Eisbrenner *et al*. 2014), the identification of multiple sex chromosomes in the masu salmon complex is a novel finding.

Hybridization within the masu salmon species complex produces fertile offspring at the same or higher rate than within species crosses (reviewed in Chevassus 1979). Hybridization can also occur in the wild when the members of the species complex overlap (Yamazaki *et al*. 2005; Kuwahara *et al*. 2019). Sex ratios in the progeny of these hybrids have not been noted. However, we would expect normal sex ratios as the location of the sdY gene would be dependent on the sire species/subspecies, and transmission of multiple sdY within an individual would not likely follow hybridization.

Previous studies have identified, however, a difference in the genomic region surrounding the sex-determining region of masu and amago (Nakayama *et al*. 1999; Zhang *et al*. 2001), and have recorded high instances of female amago with sdY genetic markers (Ueda *et al*. 2022; Hosoki *et al*. 2024). In the current study, all amago and Biwa females were sdY^−^. The difference among studies may result from using sequence data rather than amplification assays.

To understand the origins of the chromosomal diversity within Pacific salmon, synteny among the chromosomes from the different species can be used to infer fusion and fission events. Previous analyses of synteny among Pacific salmon chromosomes (Kodama *et al*. 2014; Sutherland *et al*. 2016, 2017; Blumstein *et al*. 2020) revealed relatively few conserved fusion and fission events compared to the large number of species-specific fusion events. The same trend was observed with the addition of the masu salmon (Fig. 3, Supplementary Fig. 3). There were only 4 chromosomes that did not appear to have experienced either fissions or fusions among all the Pacific salmon (Fig. 3, Supplementary Fig. 3). The fusion and fission events were inconsistent with species relatedness (e.g. pink and chum salmon shared 7 common fusion/fissions while chum salmon and rainbow trout shared 15 even though pink and chum salmon are more closely related, McKay *et al*. 1996). However, certain chromosomes with the same fusions/fissions are shared based on phylogeny (Fig. 3).

It remains unclear if there is a consequence of the variation in chromosome number and the variation in the patterns of fissions and fusions. One hypothesis is that these chromosomal fusions and fissions influence recombination and consequently the speed of adaptation (reviewed in Phillips and Ráb 2001). Another hypothesis is that rediploidization is influenced by chromosomal fusions and fissions (Sutherland *et al*. 2016). After the ancestral genome duplication in salmonids, fusions and fissions may have influenced the loss of the duplicated genome (i.e. rediploidization), which would influence the genetic diversity of a species.

### Insights from the masu (*O. m. masou*) genetic map

The initial genetic map was used to place contigs onto chromosomes. This genetic map had 43 linkage groups, which is inconsistent with the expected 33. This might be a result of using highly related broodstock to produce the genetic crosses from this study (i.e. not having enough detectable recombination events to produce the correct number of linkage groups or order markers properly). Alternatively, homologous regions are known to impact genetic map construction (Waples *et al*. 2015), and resequenced genome data from this study might produce a higher fraction of markers from these regions than traditional genetic markers. Excluding these markers or using gynogenetic haploid offspring might help in future mapping attempts (Waples *et al*. 2015). We were able to place 64% of the genome assembly onto chromosomes using this genetic map. Manual review of the Hi-C data was necessary to map more contigs and to reduce errors from placing contigs using this genetic map.

**Fig. 4.**
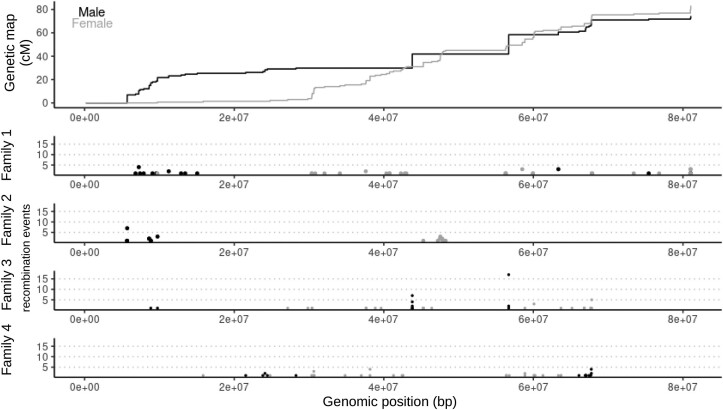
Recombination events on chromosome 1. Marey map (top), with the male (black) and the female (gray) markers plotted for the genetic map position on the *Y*-axis and the genomic position on the *X*-axis. The number of recombination events is plotted below the Marey map for each family. These correspond with the genetic map's positions. The black points are for male recombination and the gray are for female recombination.

A final genetic map was produced using the finished genome assembly, with the order of the markers from the genetic map based on their physical positions in the genome assembly. The number of linkage groups consequently matched the number of chromosomes in the genome assembly (33 linkage groups). As with other Pacific salmon, recombination events were more common in the female genetic map (Sakamoto *et al*. 2000; Matsuoka *et al*. 2004; McClelland and Naish 2008; Everett *et al*. 2012; Sutherland *et al*. 2017). Female recombination events were ∼1.25× more common in the final genetic map (Fig. 4, Supplementary Fig. 3, male genetic map: 2,045 cM, female genetic map 2,551 cM).

One hypothesis for different recombination rates between males and females of a species (known as heterochiasmy) is haploid selection (reviewed in Ritz *et al*. 2017). Haploid selection, in this context, refers to the selection that might occur at the gamete stage during the life cycle of a diploid organism. In animals, differences in male and female gametogenesis could result in recombination rate evolution due to differences in selective pressure at these stages. While only a few empirical studies have characterized the potential for haploid selection in animals (reviewed in Immler 2019), in zebrafish (*Danio rerio*), differences in gamete genotype were linked to longer-lived sperm and survival of sired embryos (Alavioon *et al*. 2017).

There was substantial variation among families in the positions of recombination events. For some linkage groups, recombination events were more common in specific locations (Supplementary Fig. 3). In other linkage groups, one of the families had much fewer recombination events. For example, family 3 had no detectable recombination events on linkage group 26 (Supplementary Fig. 3).

**Fig. 5.**
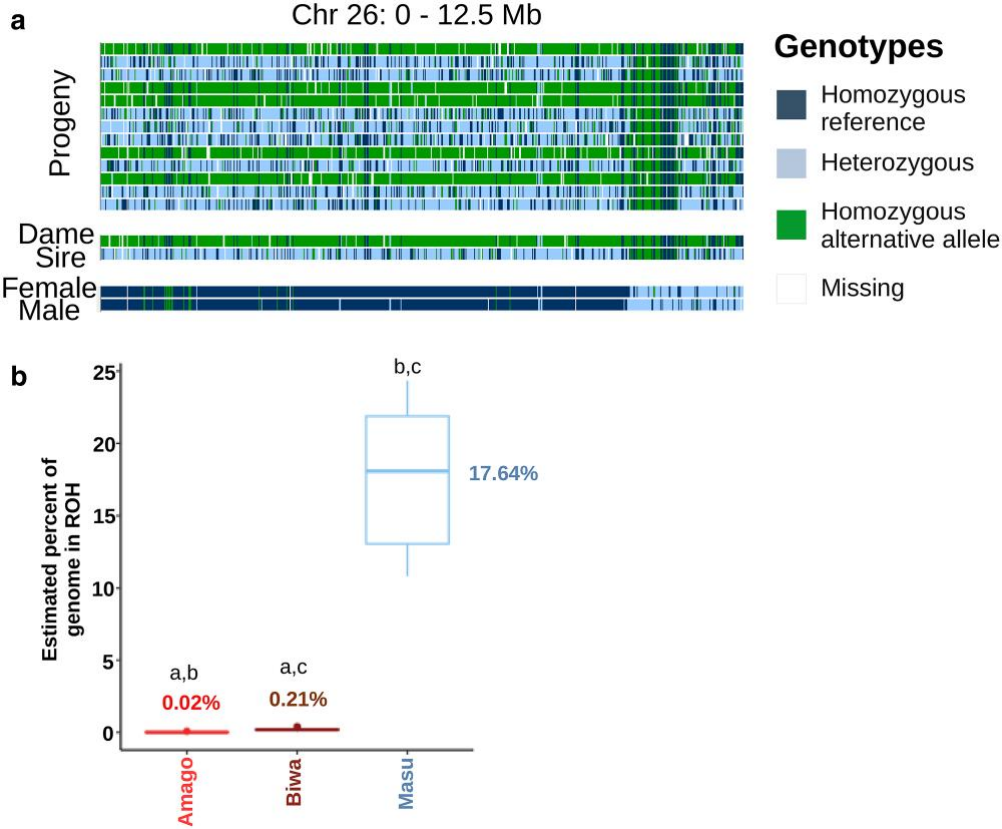
Haploblocks in broodstock of masu salmon (*O. m. masou*). a) Screenshot of half of chromosome 26 in IGV from some family 3 salmon and 2 other masu salmon broodstock. To visualize half of the chromosome, it was necessary to use the LD-filtered variants. b) Box plots of the estimated percent of the genome in runs of homozygosity (ROH) for the 3 putative subspecies. All comparisons (same letter) were significantly different (*P* ≤ 0.05) based on a 2-tailed, Welch's *t*-test.

The lack of detectable recombination might be related to the extremely long haploblocks in the masu salmon used for the reference genome assembly and the crosses used for the genetic map (Figs. 4 and 5). These salmon were from hatchery broodstock. It is clear that these salmon are inbred based on the large haploblocks and from the fraction of their genome estimated in ROH, ∼18% in masu salmon (Fig. 5).

### Variation among subspecies

**Fig. 6.**
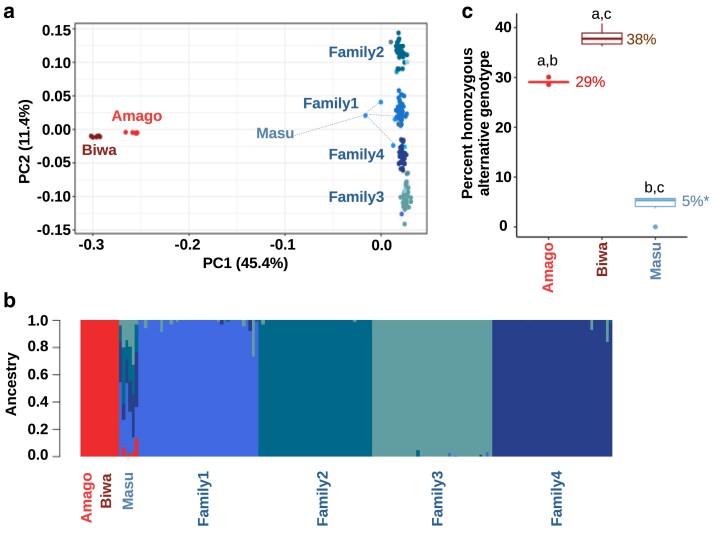
Species/subspecies principal component and admixture analyses. a) PCA with all 168 salmon with resequenced genomes. b) Bar plot of the results of an admixture analysis, with the optimal cross-validation cluster value of k = 5. c) Box plot of the percent of homozygous alternative genotypes (excludes missing genotypes) from the 3 males and 3 females of each species/subspecies. This is based on the SNPs that were not filtered based on LD (n = 13,946,349). The average value for each group is shown to the right of the box plot. The salmon that was used to polish the genome assembly was excluded from the masu salmon group (4% if included). All comparisons (same letter) were significantly different (*P* ≤ 0.05) based on a 2-tailed, Welch's *t*-test.

Amago (*O. m. ishikawae*) and Biwa salmon (*O. m.* subsp.) were distinct from the masu salmon subspecies (*O. m. masou*). This can be observed in the PCA, admixture analysis, and the number of alternative homozygous variants identified among the subspecies (Fig. 6). In the PCA, Biwa and amago salmon clustered separately but near each other on the first principal component axis (Fig. 6). This was the same pattern in a PCA when masu salmon families were removed and when all markers were used as well. However, in the other PCAs, there was more distance between Biwa and amago on the second principal component axis. In the admixture analysis, Biwa and amago salmon form 1 cluster, and the remaining clusters were based on masu salmon samples (Fig. 6).

The percent homozygous alternative genotype metric is a rough measure to quantify the percentage of the genome that differs among the subspecies. The greatest difference was between the masu and Biwa salmon with around 33% more alternative homozygous genotypes in Biwa salmon (Fig. 6). Although there is a smaller difference between Biwa and amago salmon, with 9% more alternative homozygous genotypes in Biwa salmon, this is still a significant difference (Fig. 6).

Similar to Yamamoto *et al*. (2020), the greatest genetic difference that we noted was from comparisons of *O. m. masou* to the other 2 putative subspecies. In the current study, we had fewer individuals and populations, and we did not include the *O. m. formosanus* putative subspecies. Because of these limitations, inferences on how we might categorize the species complex are limited. What we can start to infer is the scale of these differences. The sampled *O. m. ishikawae* and *O. m.* subsp. genomes had around 29–38% homozygous alternative alleles relative to the *O. m. masou* reference genome assembly, respectively. Further sampling will reveal if this phenomenon is an artifact of sampling bias, or if indeed around a third of the genetic variants in the masu salmon species complex are different among the suspected subspecies.

## Conclusions

With this study, all of the Pacific salmon now have a reference genome assembly (Christensen *et al*. 2018; Christensen *et al*. 2020; Christensen *et al*. 2021; Rondeau *et al*. 2023a; Rondeau *et al*. 2023b). This facilitates the transfer of information from other Pacific salmon and unrelated species. Among other insights, the present study uncovered that the sex chromosome in at least 1 possible subspecies of the masu salmon complex is unique among Pacific salmon. Future studies will examine genes in masu salmon known to impact valuable traits in other Pacific salmon, and the reference genome assembly will aid in these studies.

Genome assemblies of the other species/subspecies would be effective in identifying any major structural changes among potential subspecies of masu salmon. Structural differences could be a practical criterion in determining classification as subspecies or species. The limited resequencing from this study quantified that around one-third of the SNPs in the amago and Biwa were homozygous alternative genotypes to the reference genome assembly. This information may also be suitable for management and classification purposes.

## Supplementary Material

jkae278_Supplementary_Data

## Data Availability

Sequencing data is associated with NCBI BioProject: PRJNA999701. The Python script used in this study can be found at github.com/KrisChristensen/VCFstats. Supplemental material available at G3 online.
