# Supplementary material for: Masu salmon species complex relationships and sex chromosomes revealed from analyses of the masu salmon (Oncorhynchus masou masou) genome assembly
Source: G3 (Bethesda). 2024 Nov 28;15(2):jkae278. doi: 10.1093/g3journal/jkae278 (PMC11797027; doi:10.1093/g3journal/jkae278)
Supplement: jkae278_Supplementary_Data [file jkae278_supplementary_data.zip › Figure_S3_G3-2024-405504.pdf]

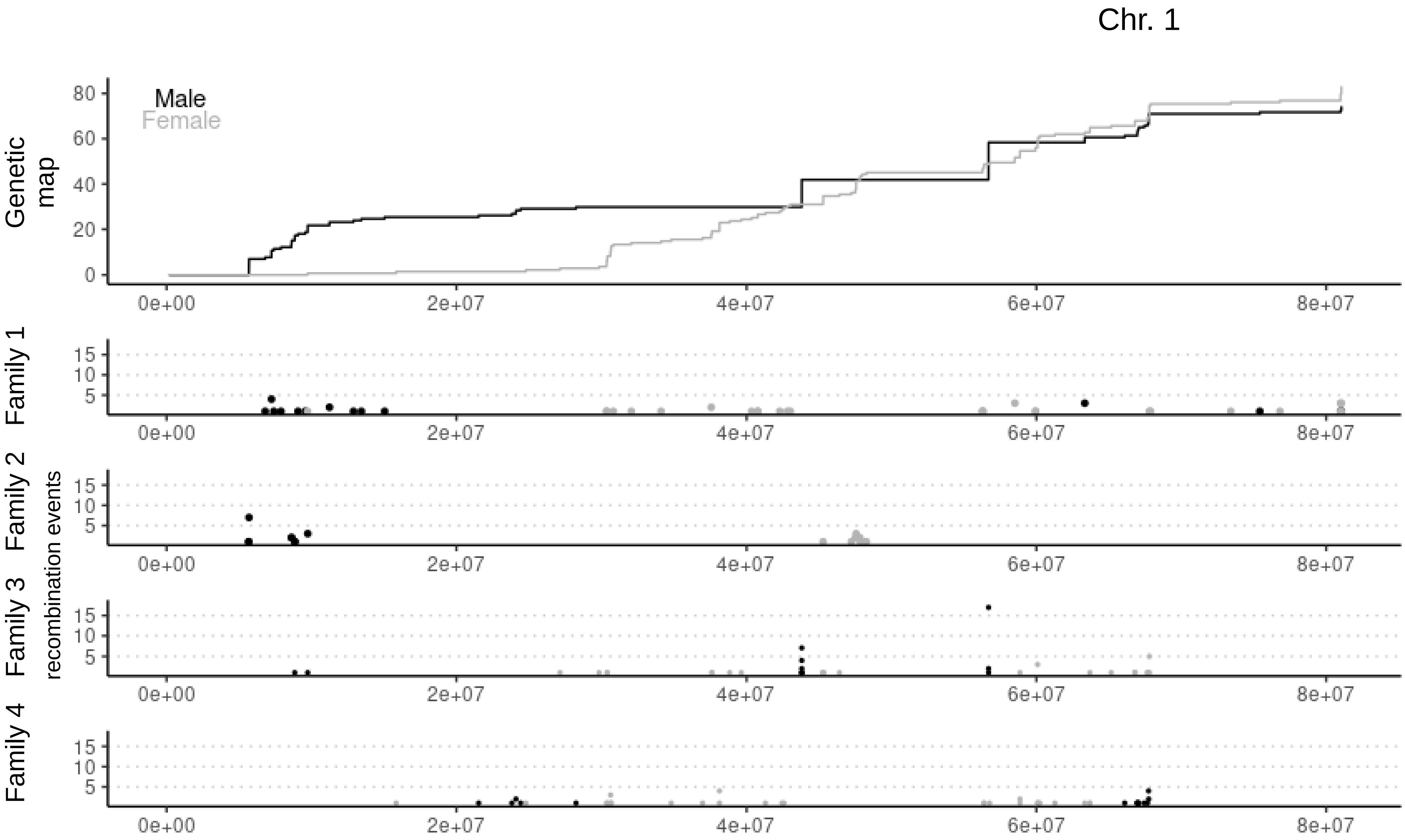

Chr. 1
Male
Female
Genetic map
80
60
40
20
0
0e+00
2e+07
4e+07
6e+07
8e+07
Family 1
Family 2
Family 3
Family 4
recombination events
15
10
5

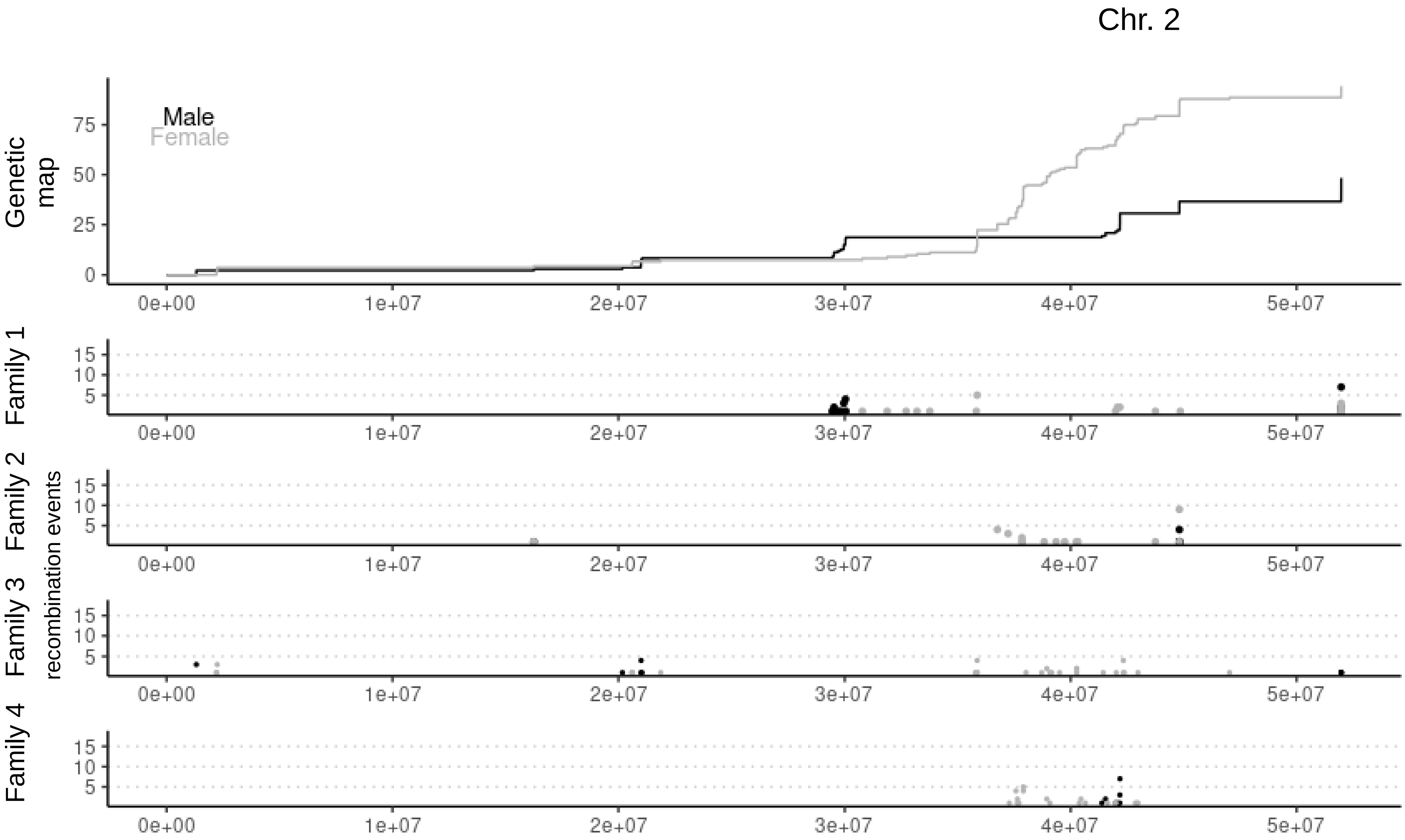
Chr. 2
Male
Female
Genetic map
0
25
50
75
0e+00
1e+07
2e+07
3e+07
4e+07
5e+07
Family 1
Family 2
Family 3
Family 4
recombination events
5
10
15

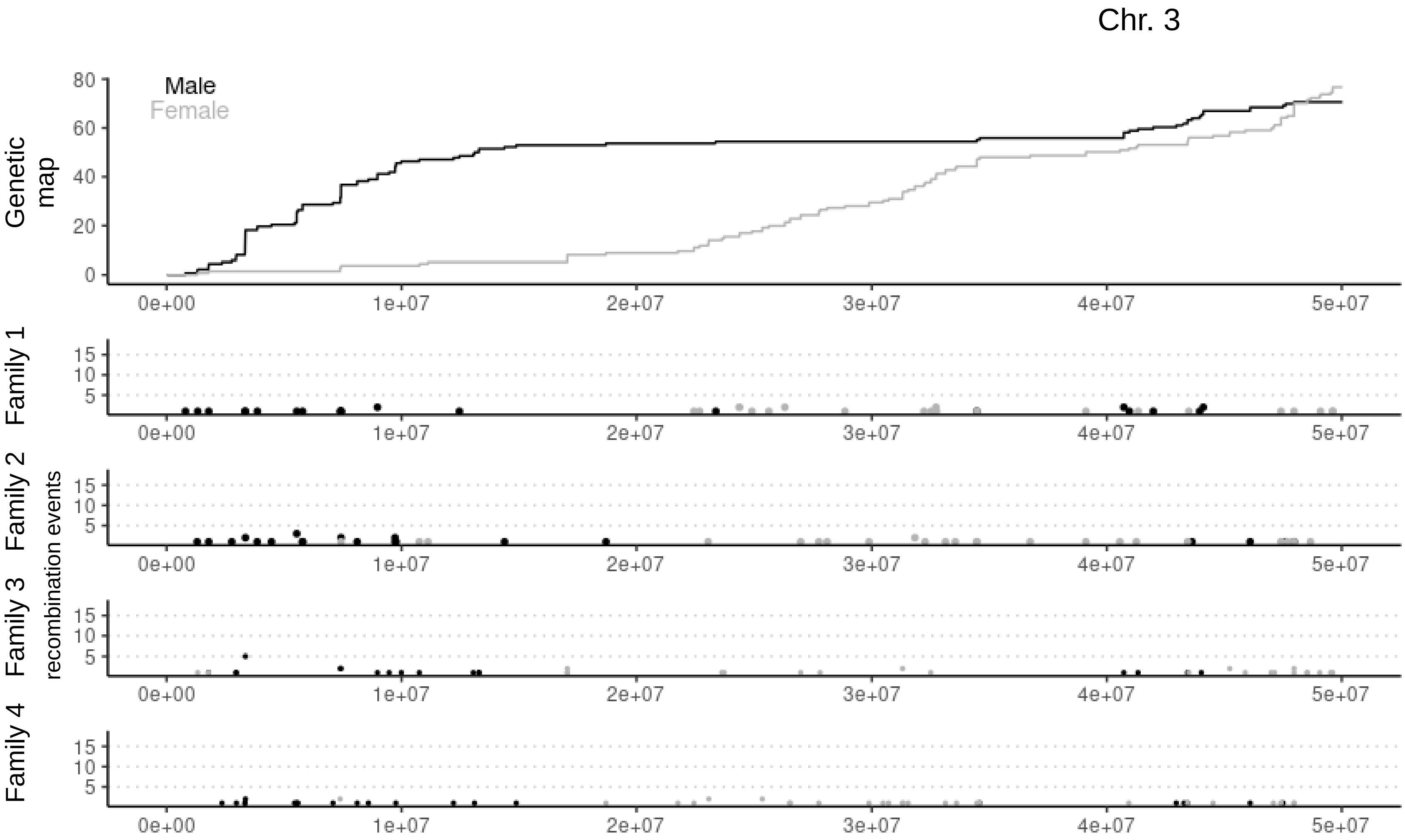

Chr. 3
Male
Female
Genetic map
80
60
40
20
0
0e+00
1e+07
2e+07
3e+07
4e+07
5e+07
Family 1
Family 2
Family 3
Family 4
recombination events
15
10
5

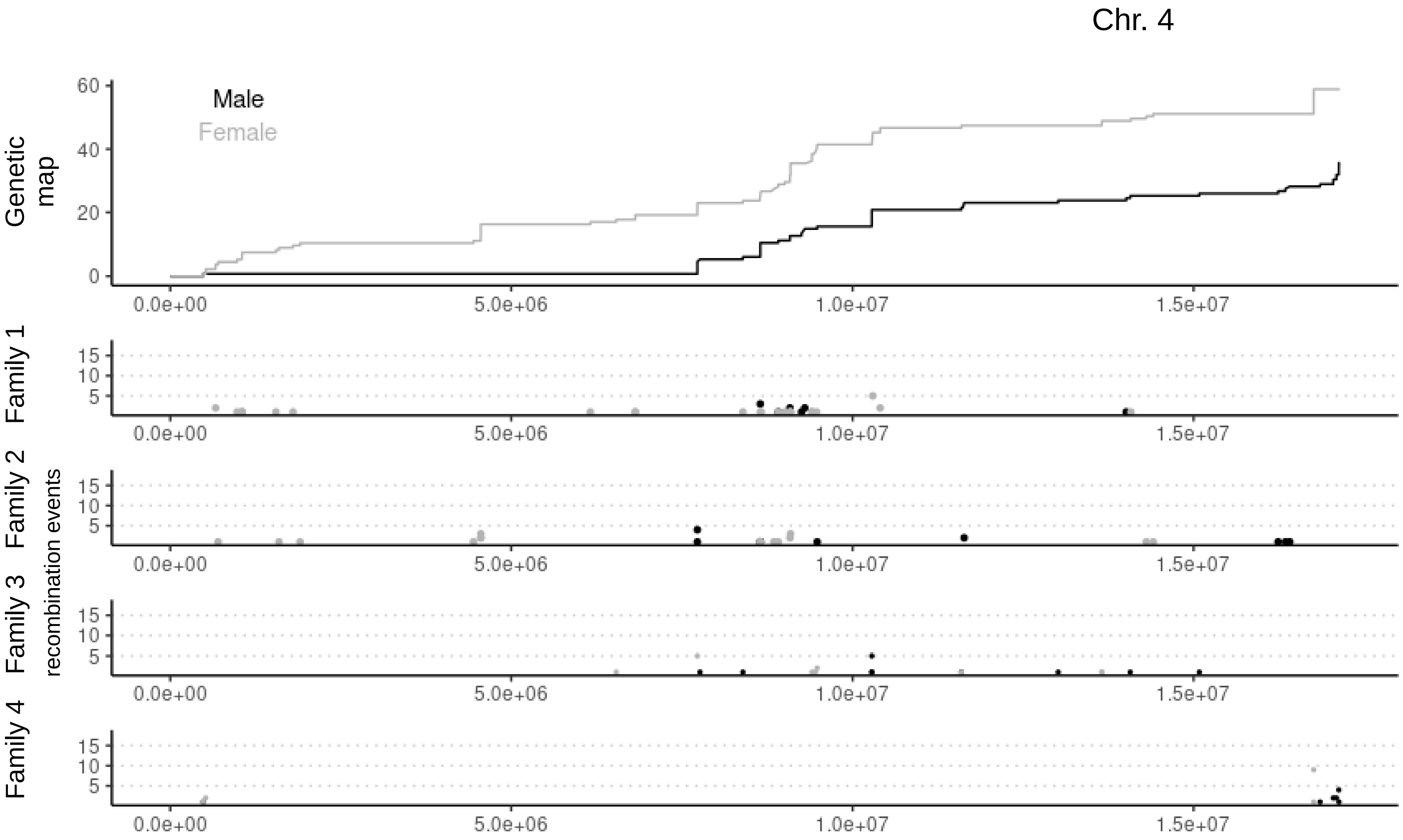
Chr. 4
Male
Female
Genetic map
60
40
20
0
0.0e+00
5.0e+06
1.0e+07
1.5e+07
Family 1
Family 2
Family 3
Family 4
recombination events
15
10
5

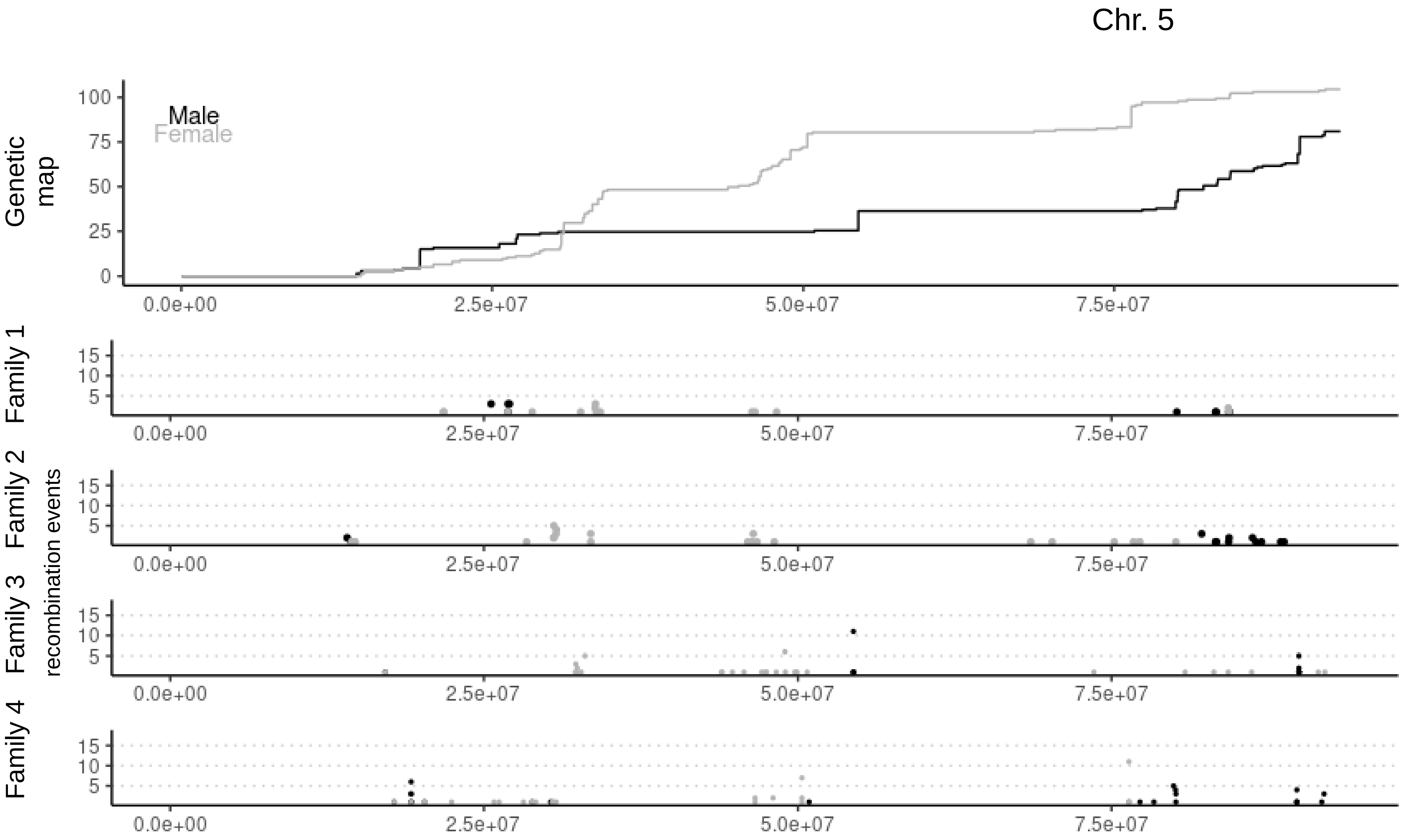
Chr. 5
Genetic map
Male
Female
100
75
50
25
0
0.0e+00
2.5e+07
5.0e+07
7.5e+07
Family 1
Family 2
Family 3
Family 4
recombination events
15
10
5

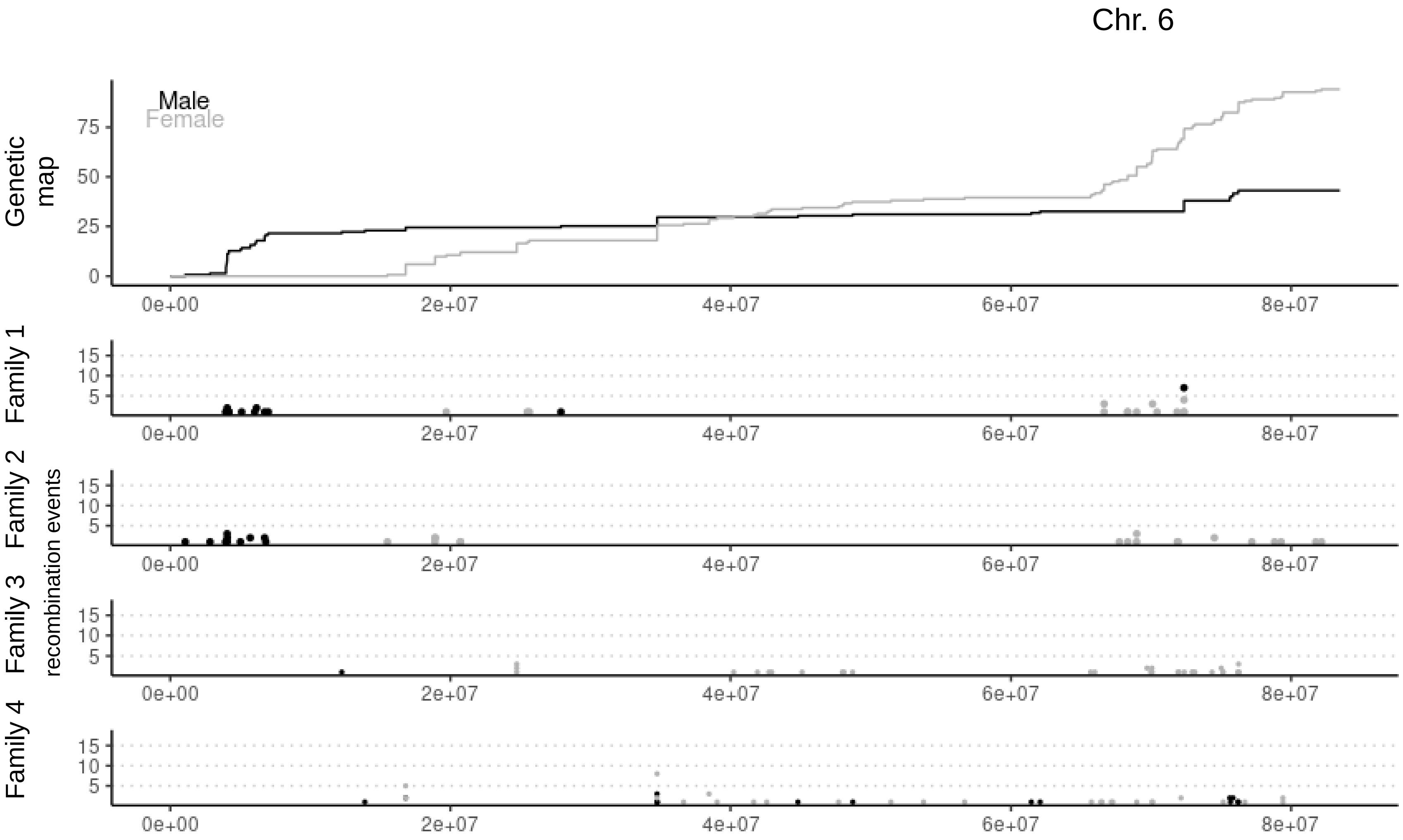
Chr. 6
Male
Female
Genetic map
0
25
50
75
0e+00
2e+07
4e+07
6e+07
8e+07
Family 1
Family 2
Family 3
Family 4
recombination events
5
10
15

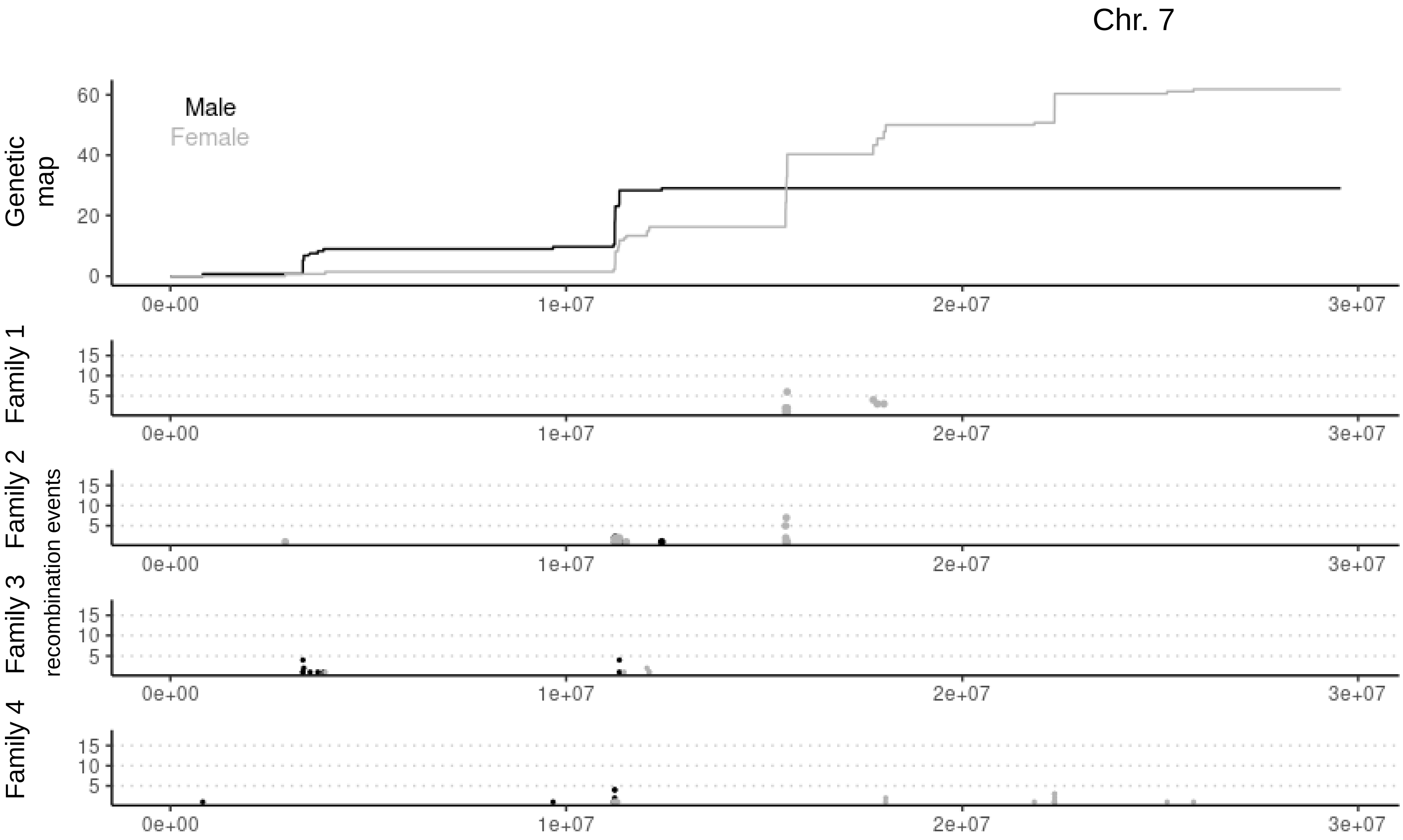

Chr. 7
Male
Female
Genetic map
0
20
40
60
Family 1
Family 2
Family 3
Family 4
recombination events
5
10
15
0e+00
1e+07
2e+07
3e+07

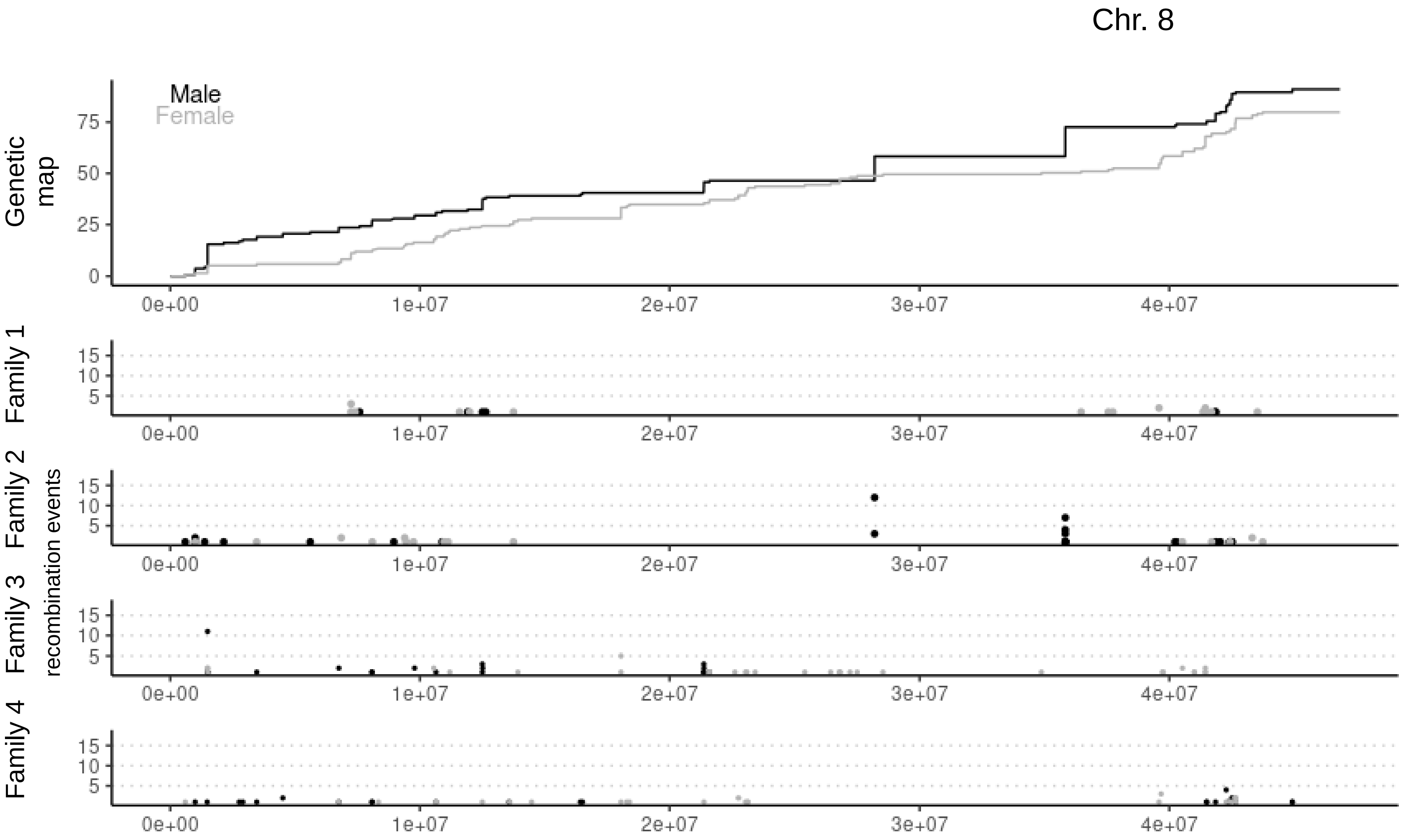

Chr. 8
Male
Female
Genetic map
0
25
50
75
0e+00
1e+07
2e+07
3e+07
4e+07
Family 1
Family 2
Family 3
Family 4
recombination events
5
10
15

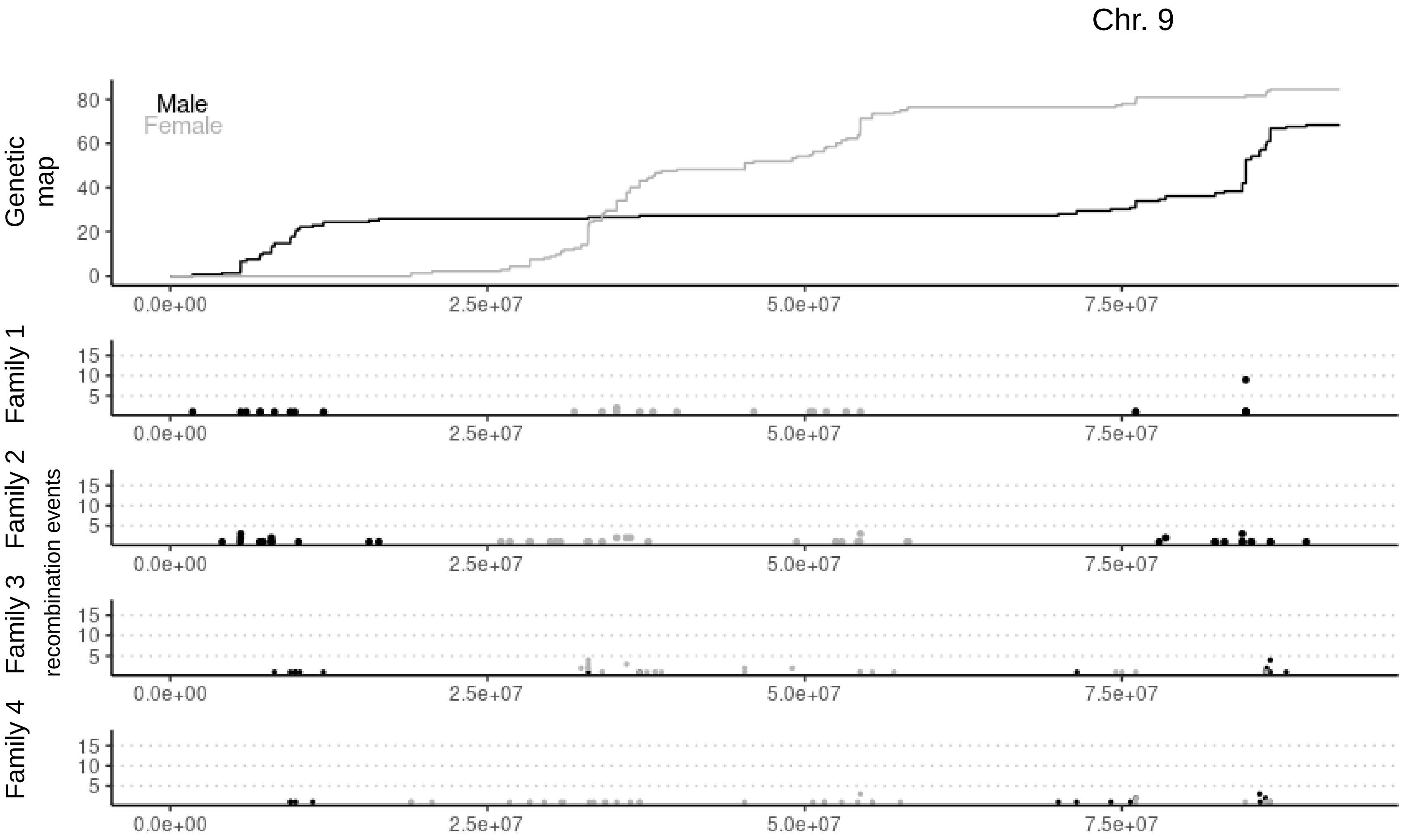
Chr. 9
Genetic map
Male
Female
80
60
40
20
0
0.0e+00
2.5e+07
5.0e+07
7.5e+07
Family 1
Family 2
Family 3
Family 4
recombination events
15
10
5

Chr. 10

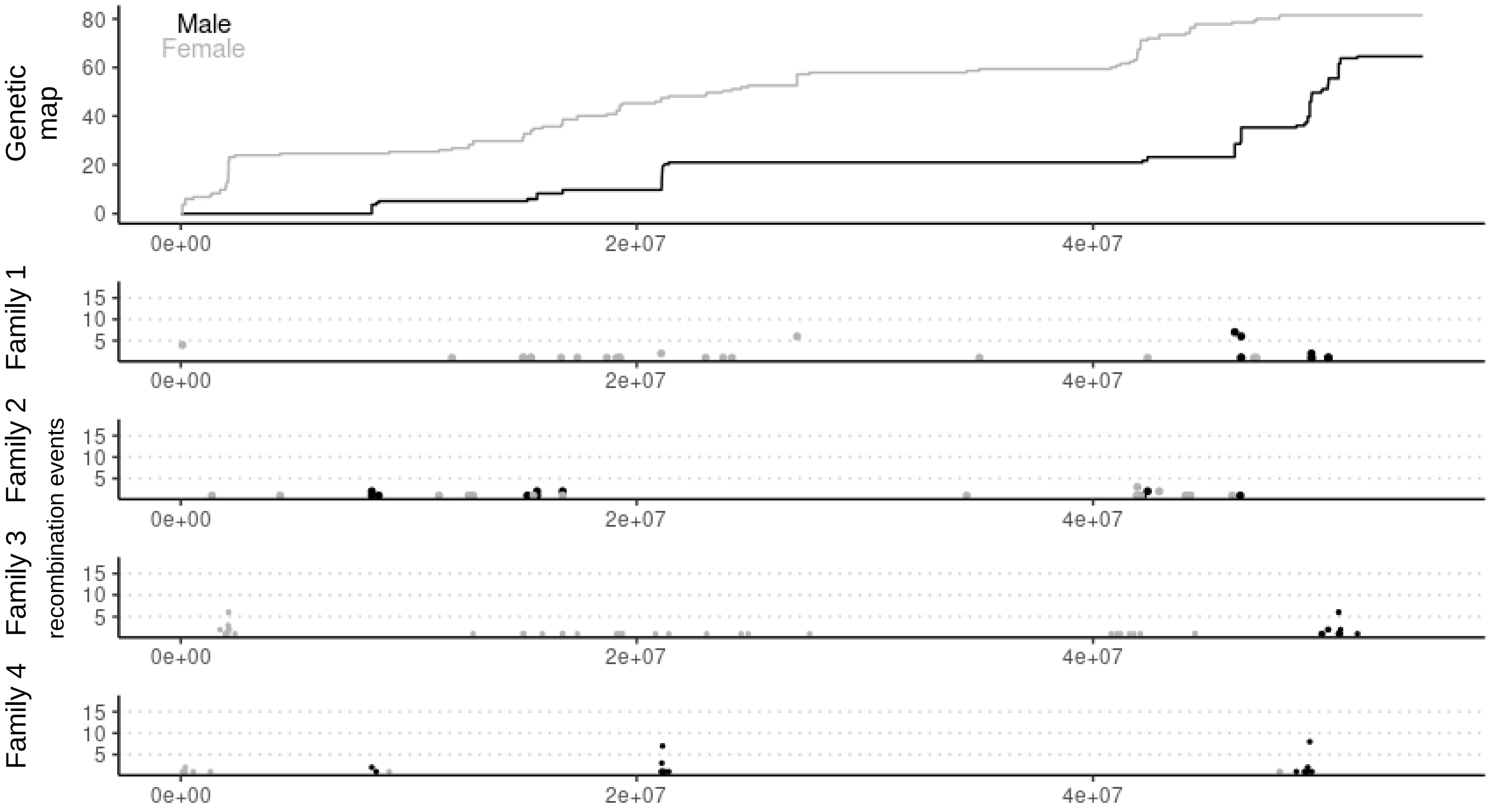
Male
Female
Genetic map
80
60
40
20
0
0e+00
2e+07
4e+07
Family 1
Family 2
Family 3
Family 4
recombination events
15
10
5

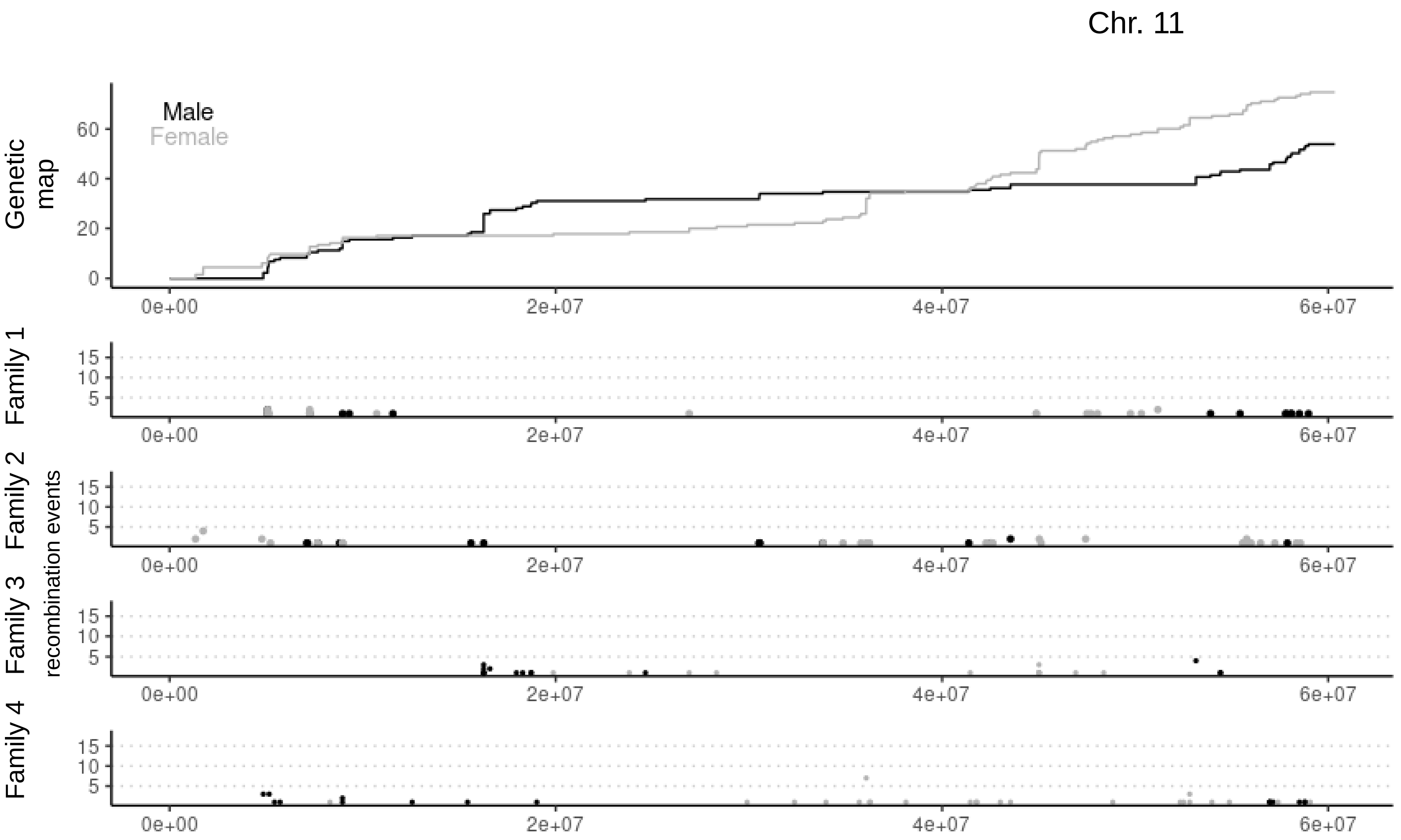
Chr. 11
Male
Female
Genetic map
60
40
20
0
0e+00
2e+07
4e+07
6e+07
Family 1
Family 2
Family 3
Family 4
15
10
5
recombination events

Chr. 12

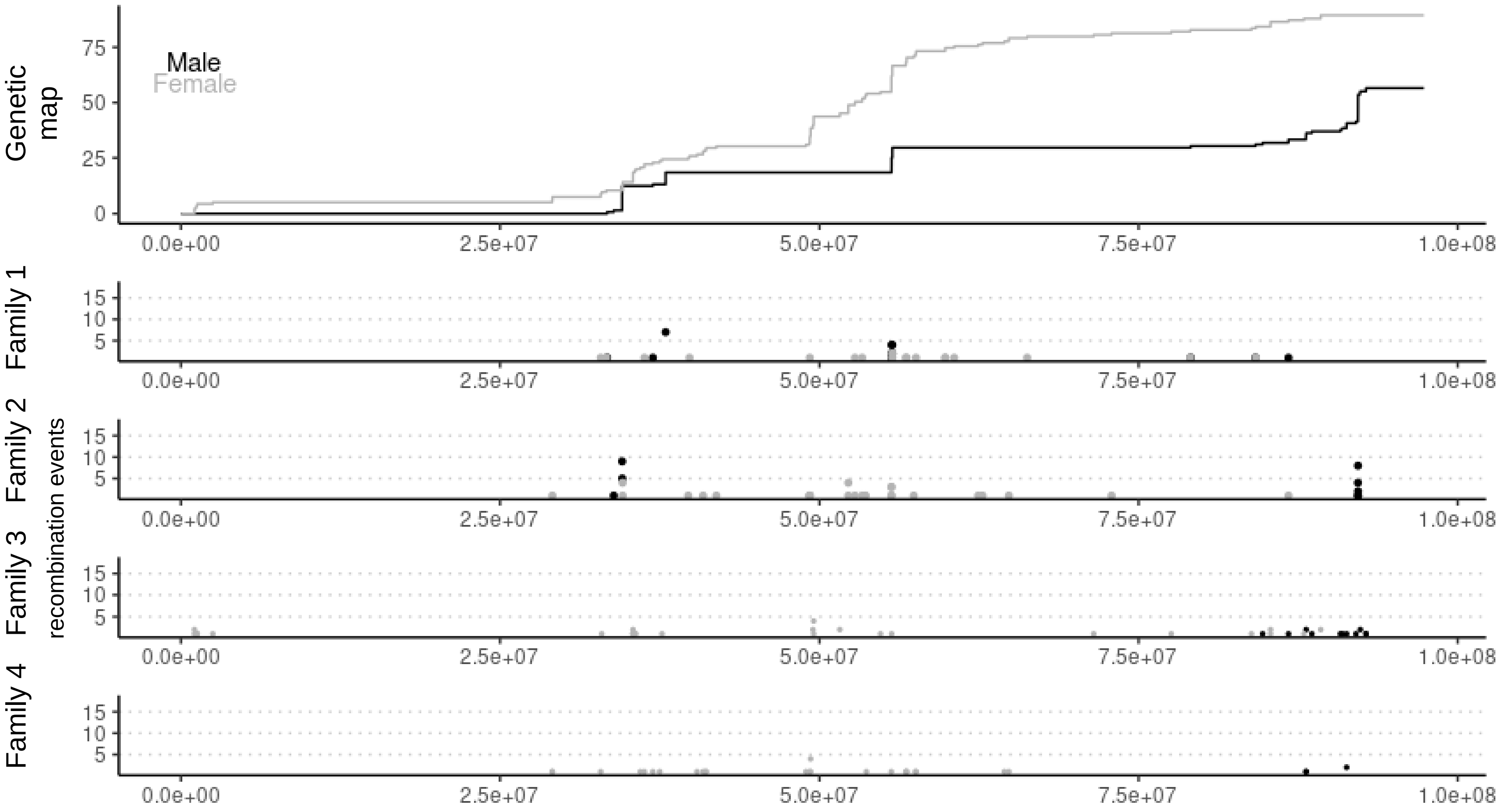
Genetic map
Male
Female
75
50
25
0
0.0e+00
2.5e+07
5.0e+07
7.5e+07
1.0e+08
Family 1
Family 2
Family 3
Family 4
recombination events
15
10
5

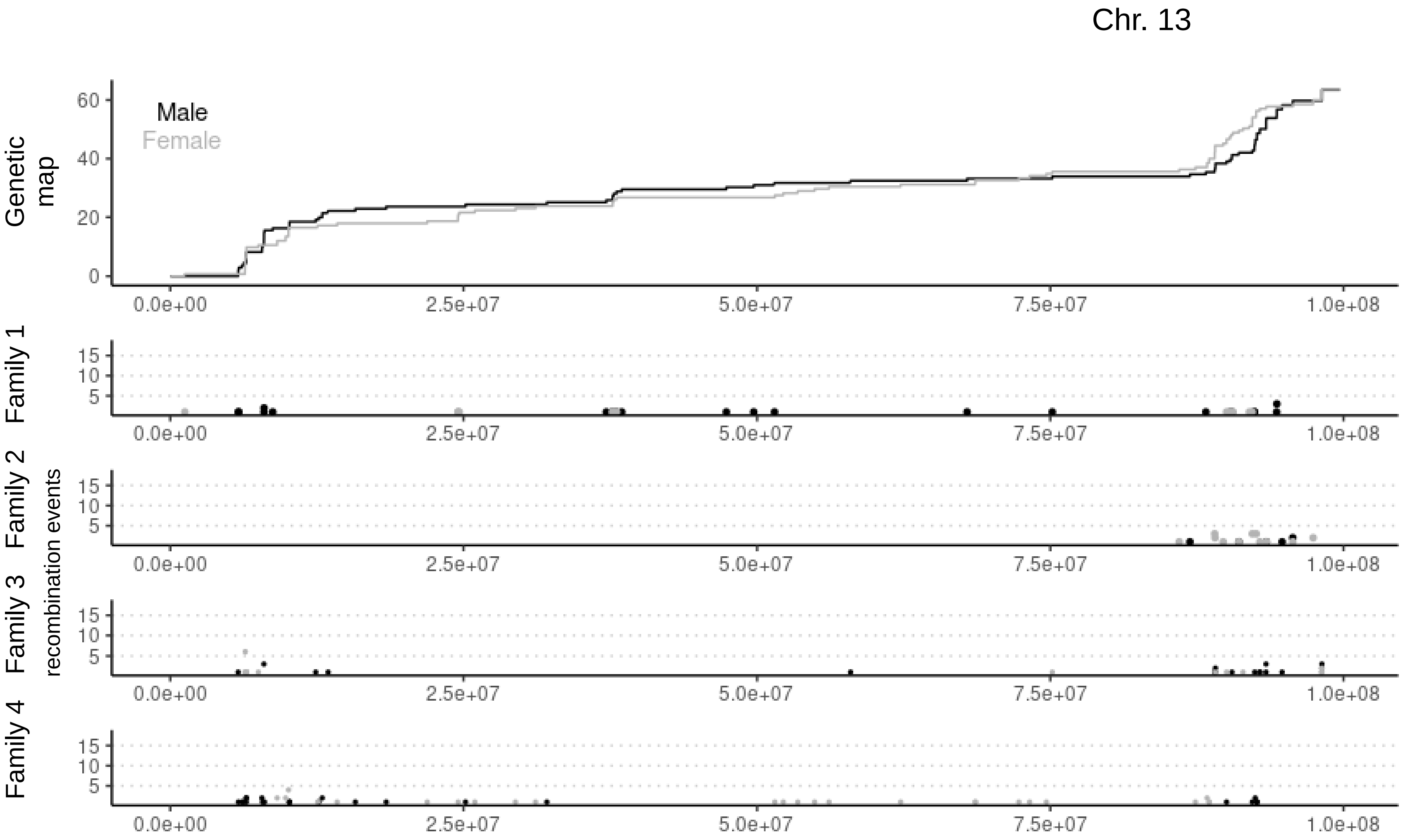
Chr. 13
Genetic map
Male
Female
60
40
20
0
0.0e+00
2.5e+07
5.0e+07
7.5e+07
1.0e+08
Family 1
Family 2
Family 3
Family 4
recombination events
15
10
5

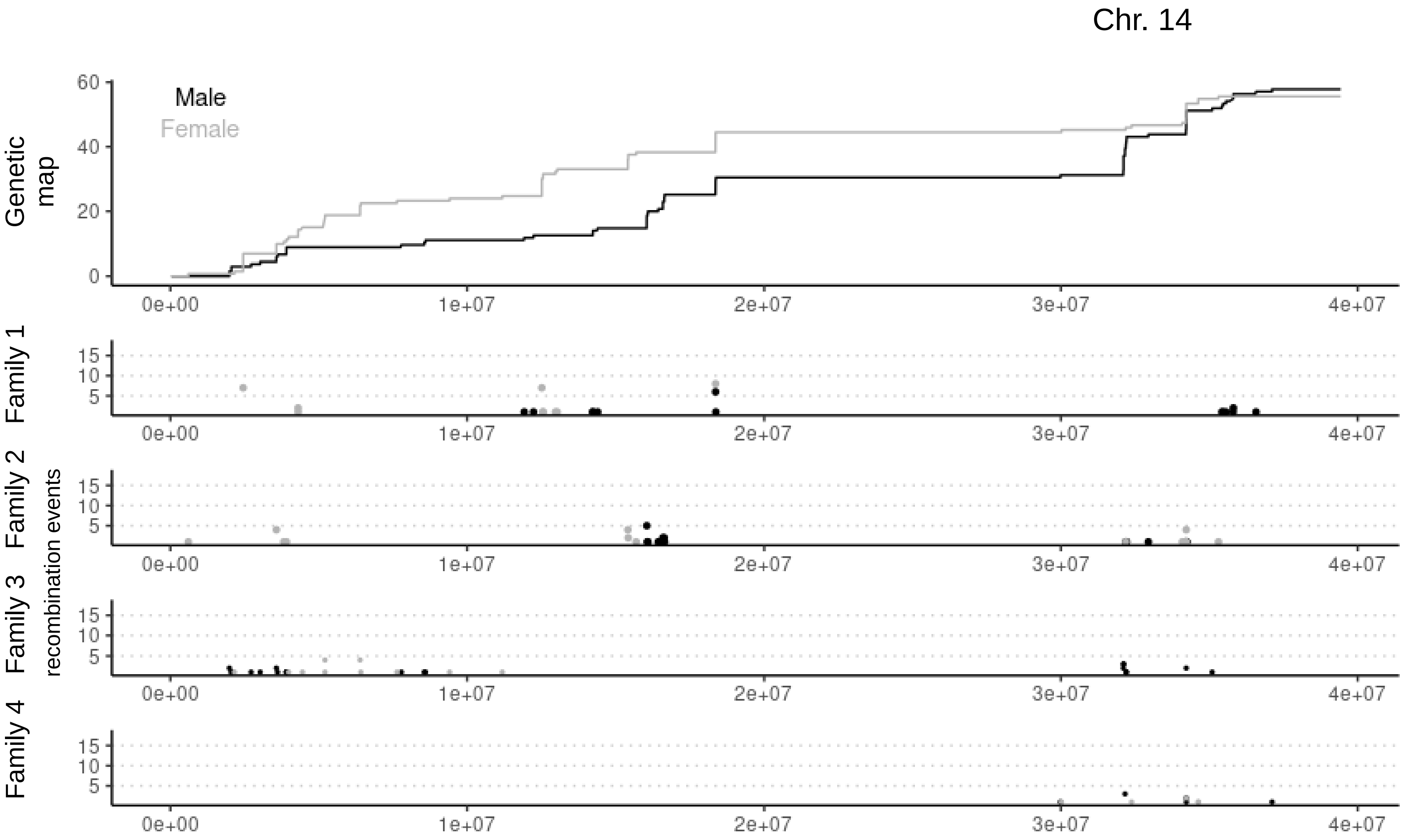
Chr. 14
Male
Female
Genetic map
60
40
20
0
0e+00
1e+07
2e+07
3e+07
4e+07
Family 1
Family 2
Family 3
Family 4
recombination events
15
10
5

Chr. 15

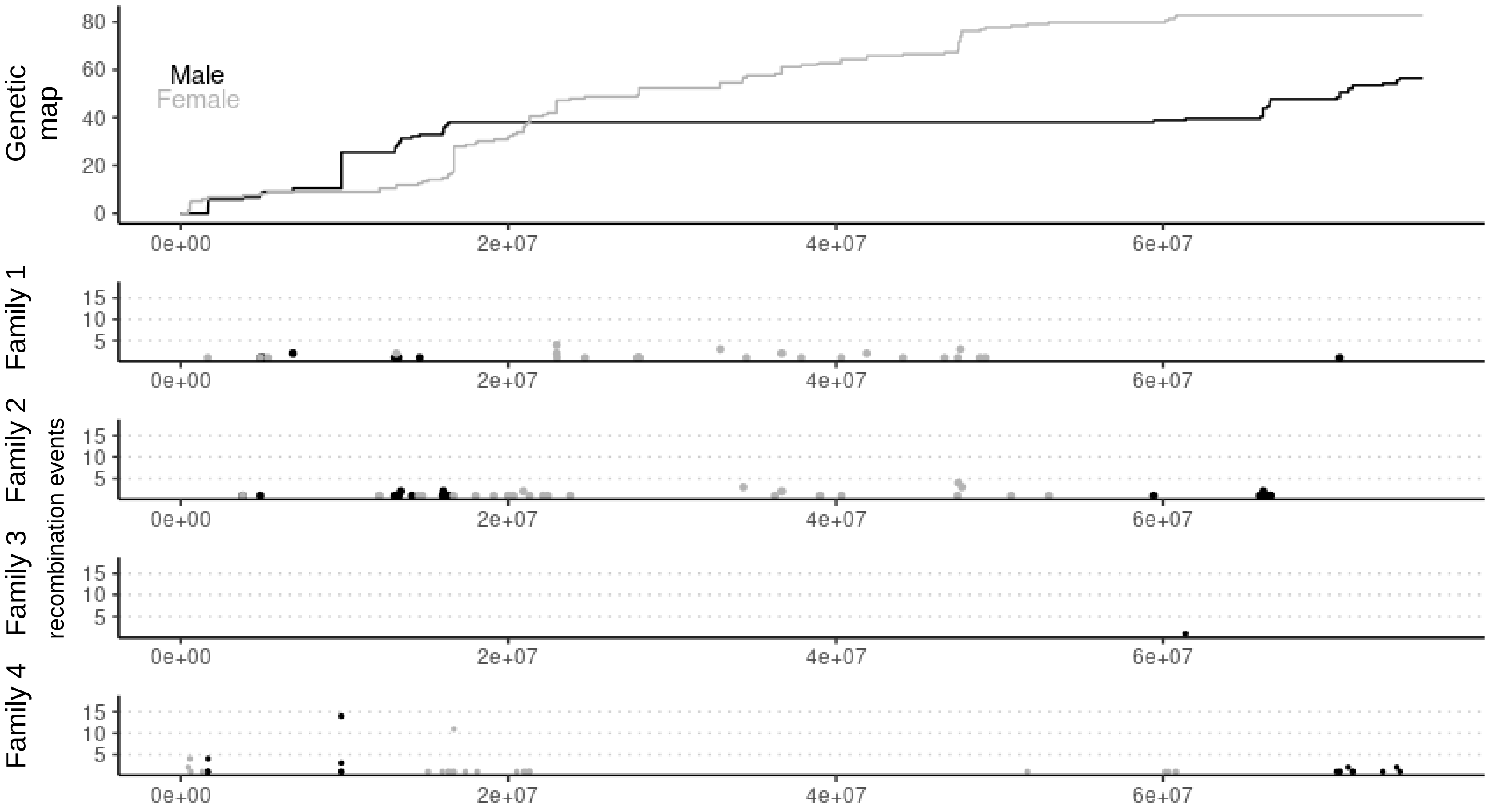

Genetic map
Male
Female
80
60
40
20
0
0e+00
2e+07
4e+07
6e+07
Family 1
Family 2
Family 3
Family 4
recombination events
15
10
5

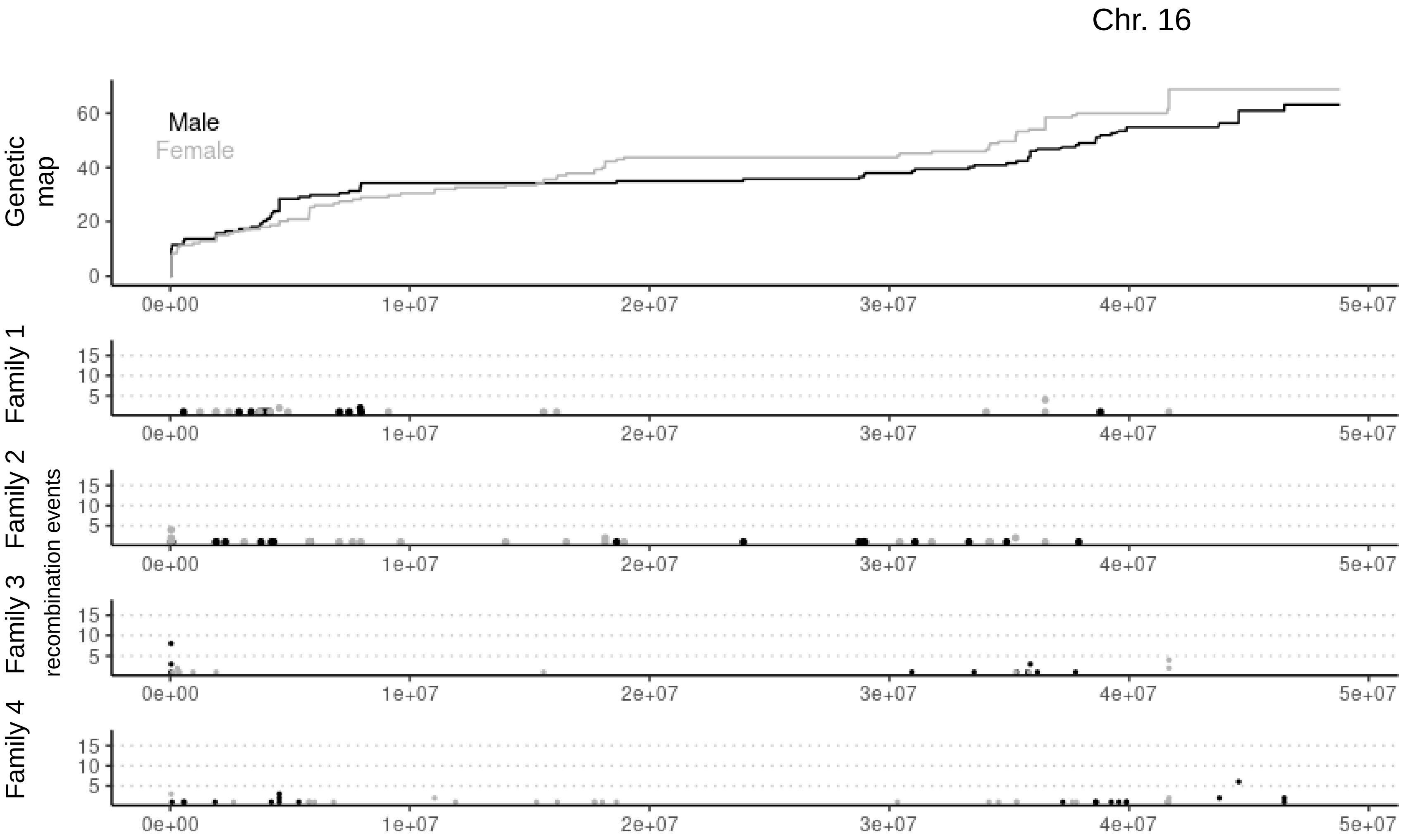
Chr. 16
Genetic map
Male
Female
60
40
20
0
0e+00
1e+07
2e+07
3e+07
4e+07
5e+07
Family 1
Family 2
Family 3
Family 4
recombination events
15
10
5

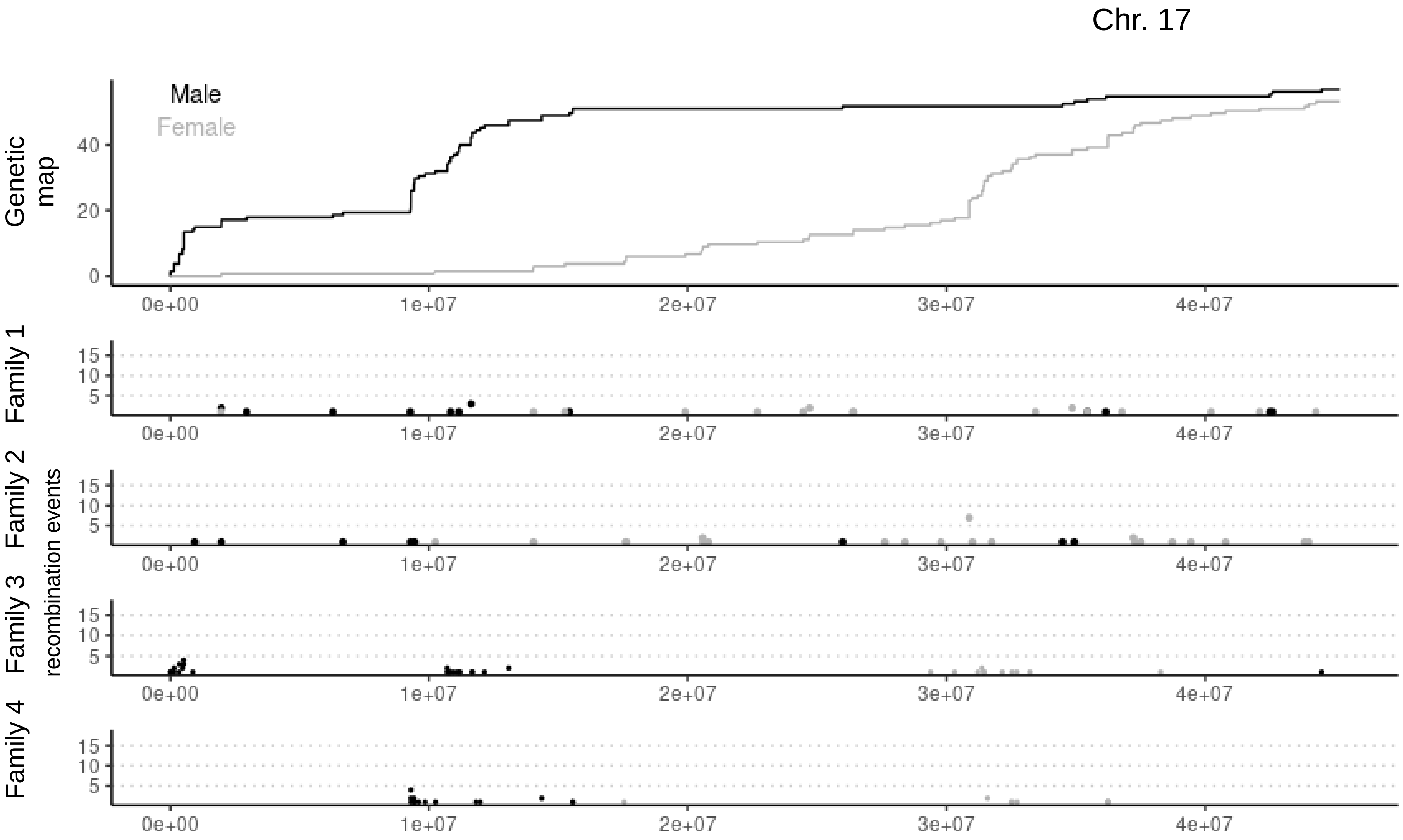

Chr. 17
Male
Female
Genetic map
Family 1
Family 2
Family 3
Family 4
recombination events
0
20
40
5
10
15
0e+00
1e+07
2e+07
3e+07
4e+07

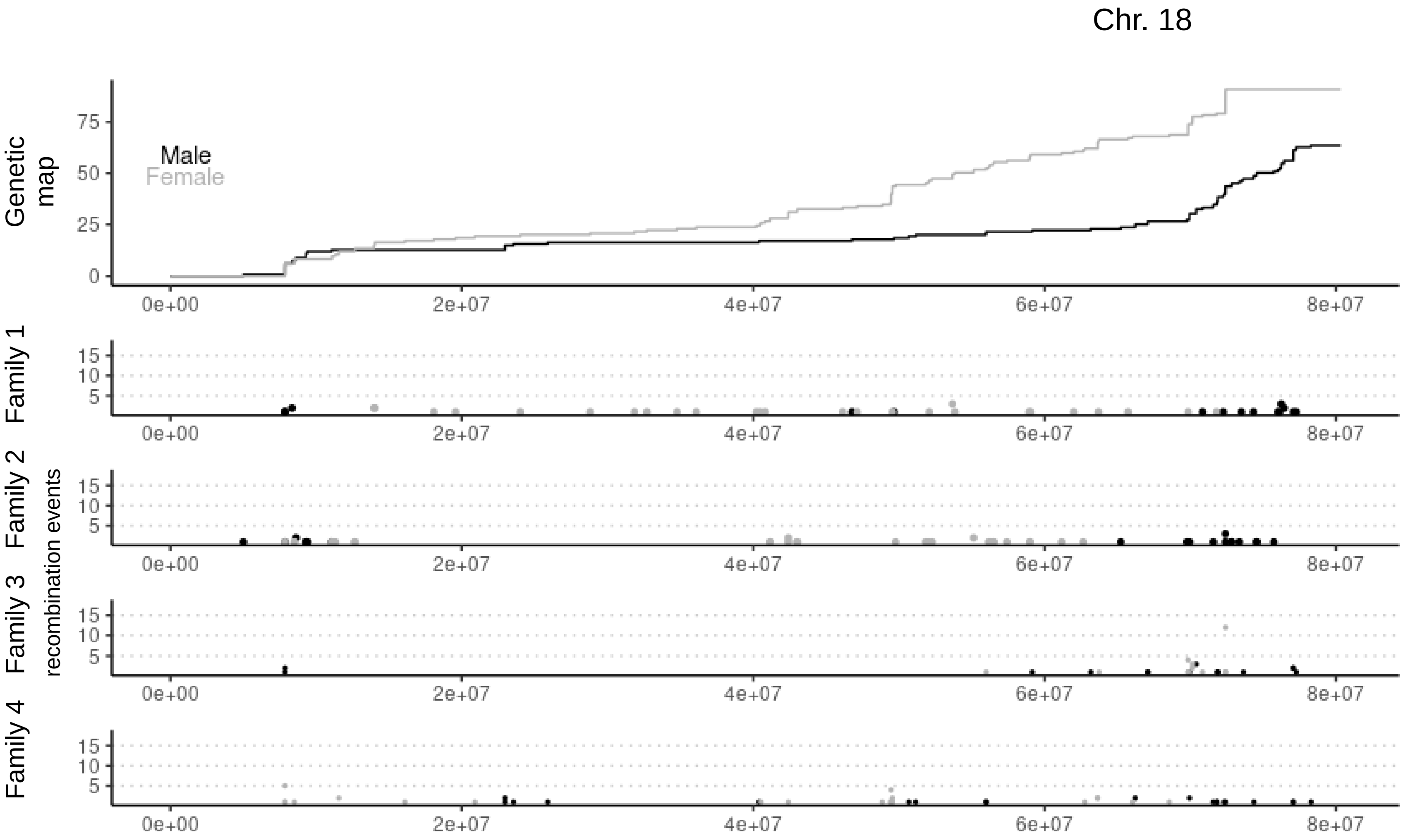
Chr. 18
Genetic map
Male
Female
75
50
25
0
0e+00
2e+07
4e+07
6e+07
8e+07
Family 1
Family 2
Family 3
Family 4
recombination events
15
10
5

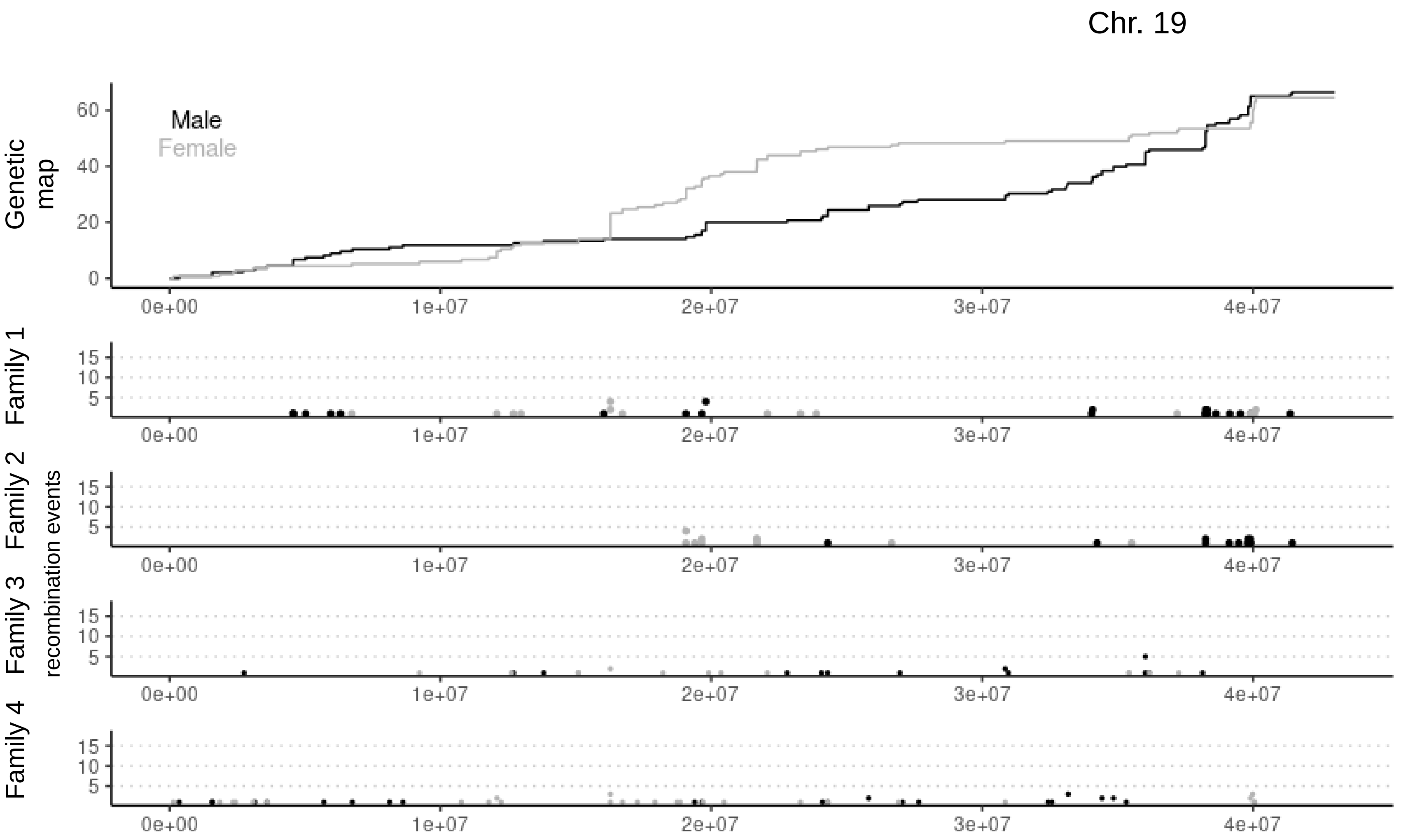
Chr. 19
Genetic map
Male
Female
60
40
20
0
0e+00
1e+07
2e+07
3e+07
4e+07
Family 1
Family 2
Family 3
Family 4
recombination events
15
10
5

Chr. 20

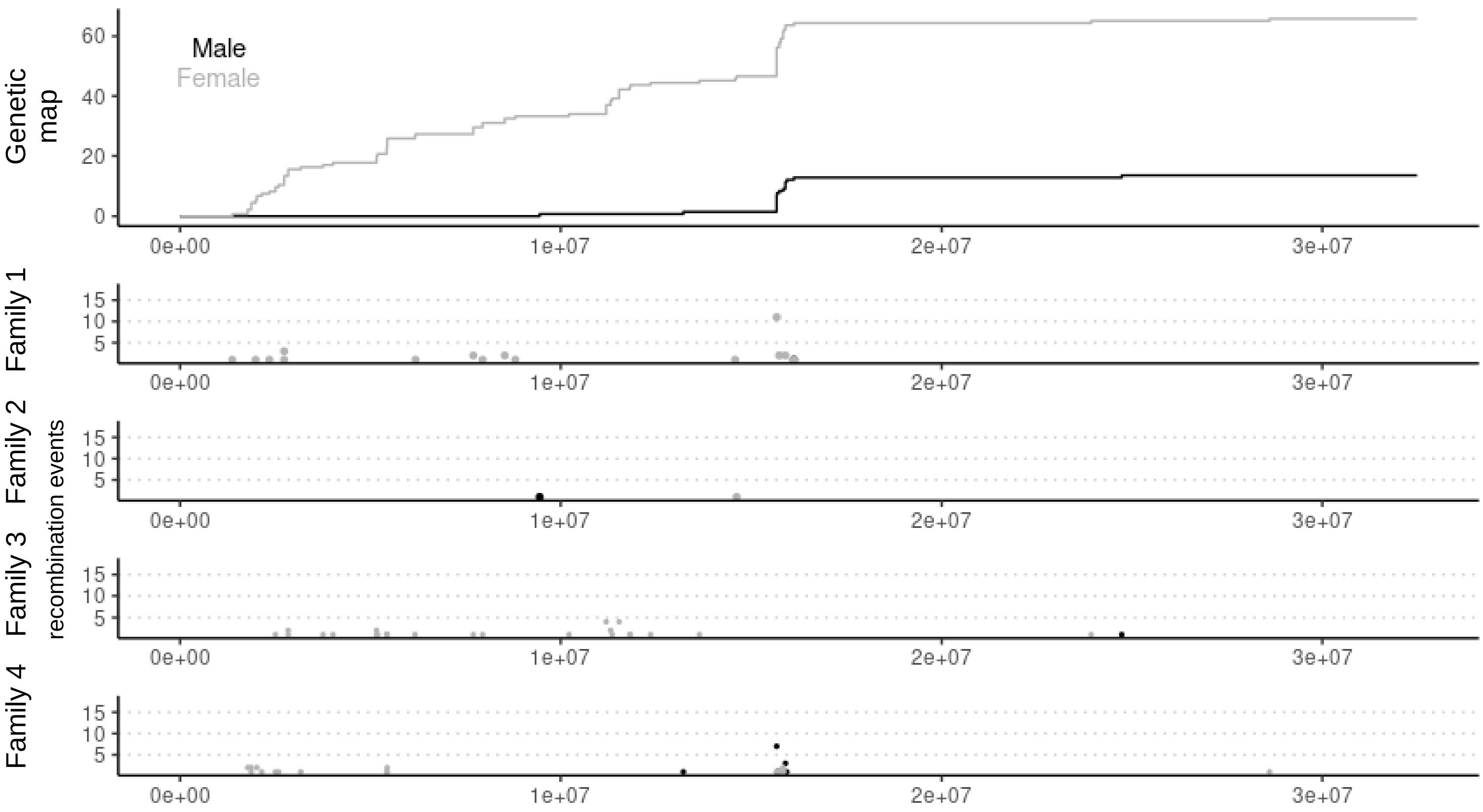
Genetic map
Male
Female
60
40
20
0
0e+00
1e+07
2e+07
3e+07
Family 1
Family 2
Family 3
Family 4
recombination events
15
10
5

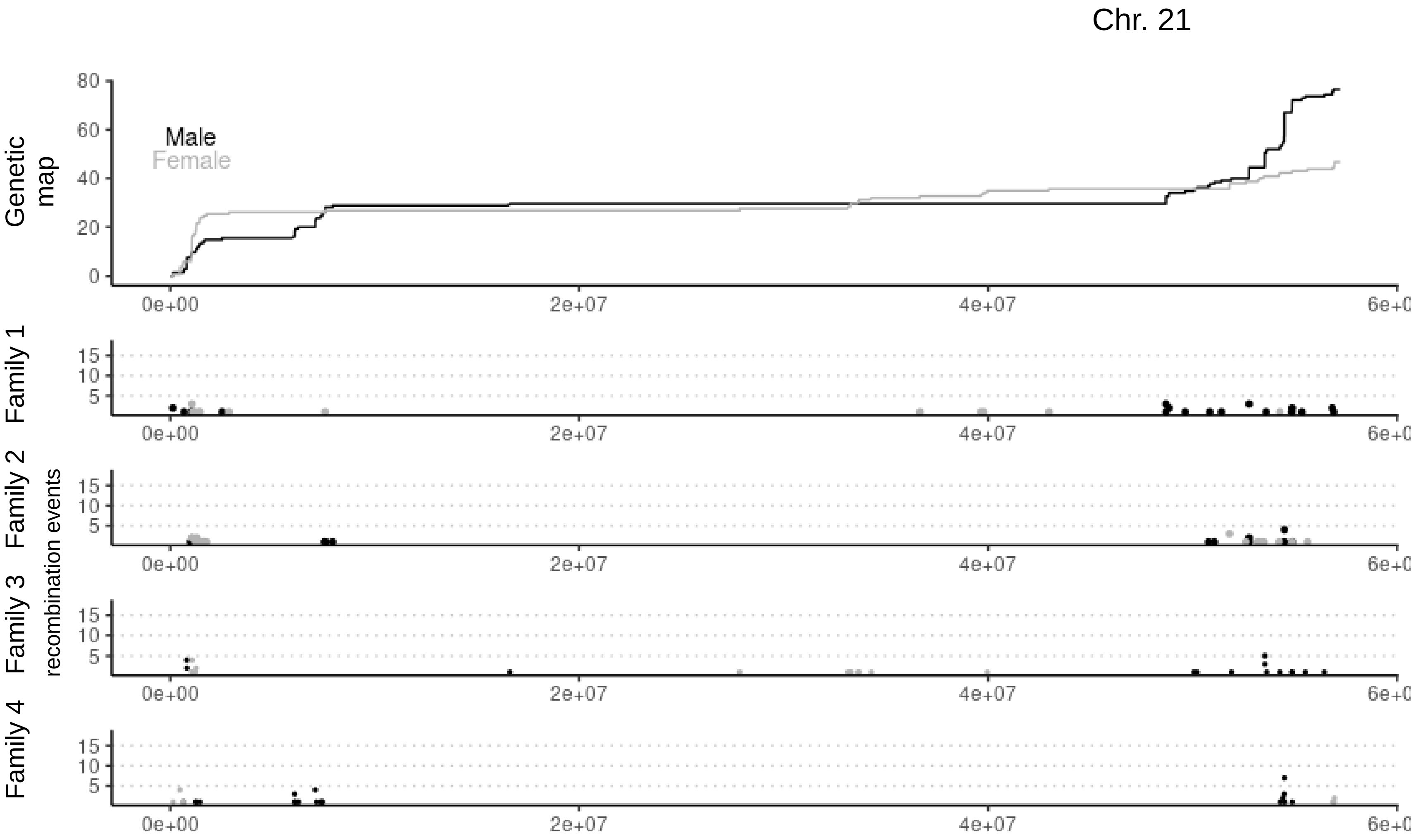

Chr. 21
Genetic map
Male
Female
80
60
40
20
0
0e+00
2e+07
4e+07
6e+0
Family 1
Family 2
Family 3
Family 4
recombination events
15
10
5

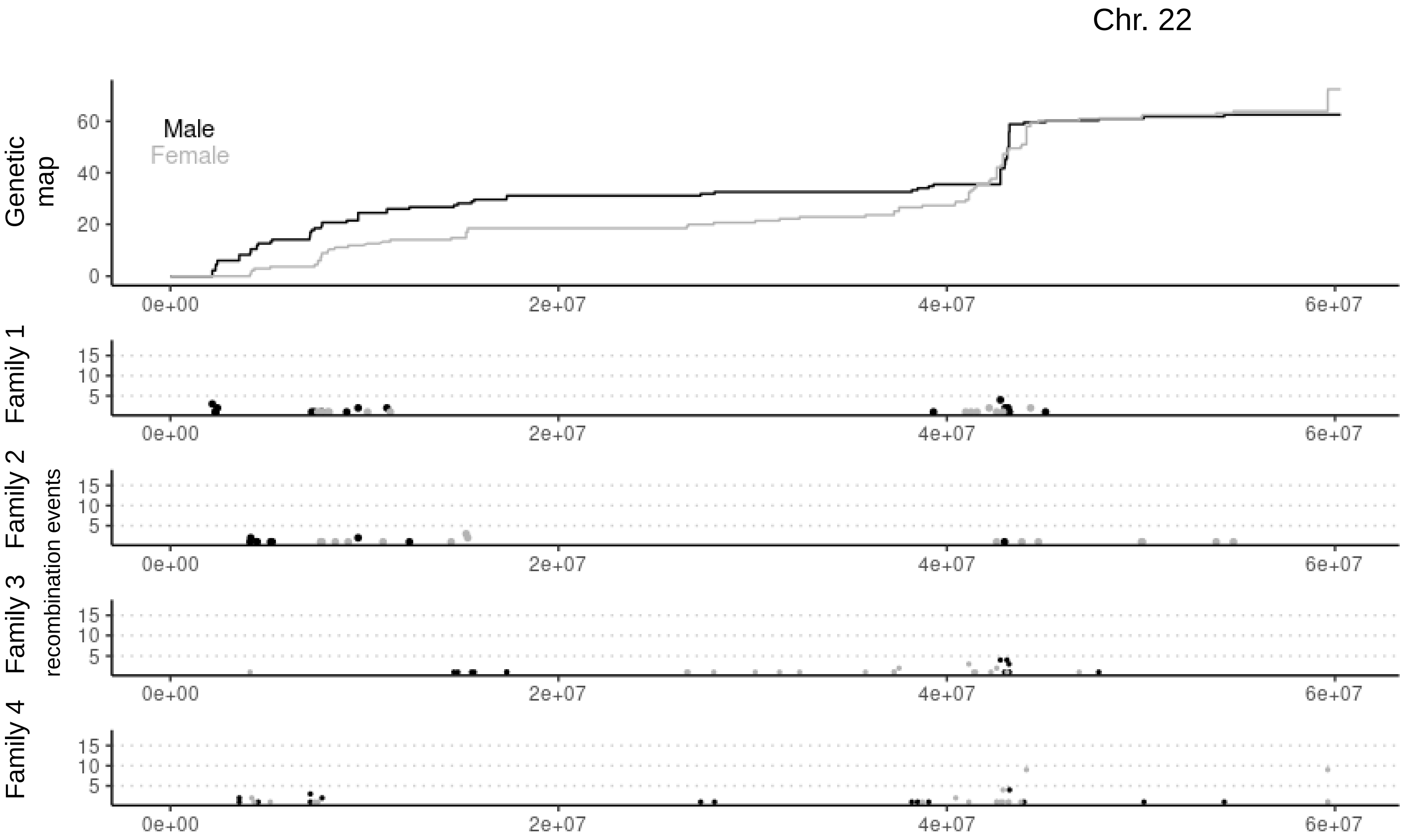
Chr. 22
Genetic map
Male
Female
60
40
20
0
0e+00
2e+07
4e+07
6e+07
Family 1
Family 2
Family 3
Family 4
recombination events
15
10
5

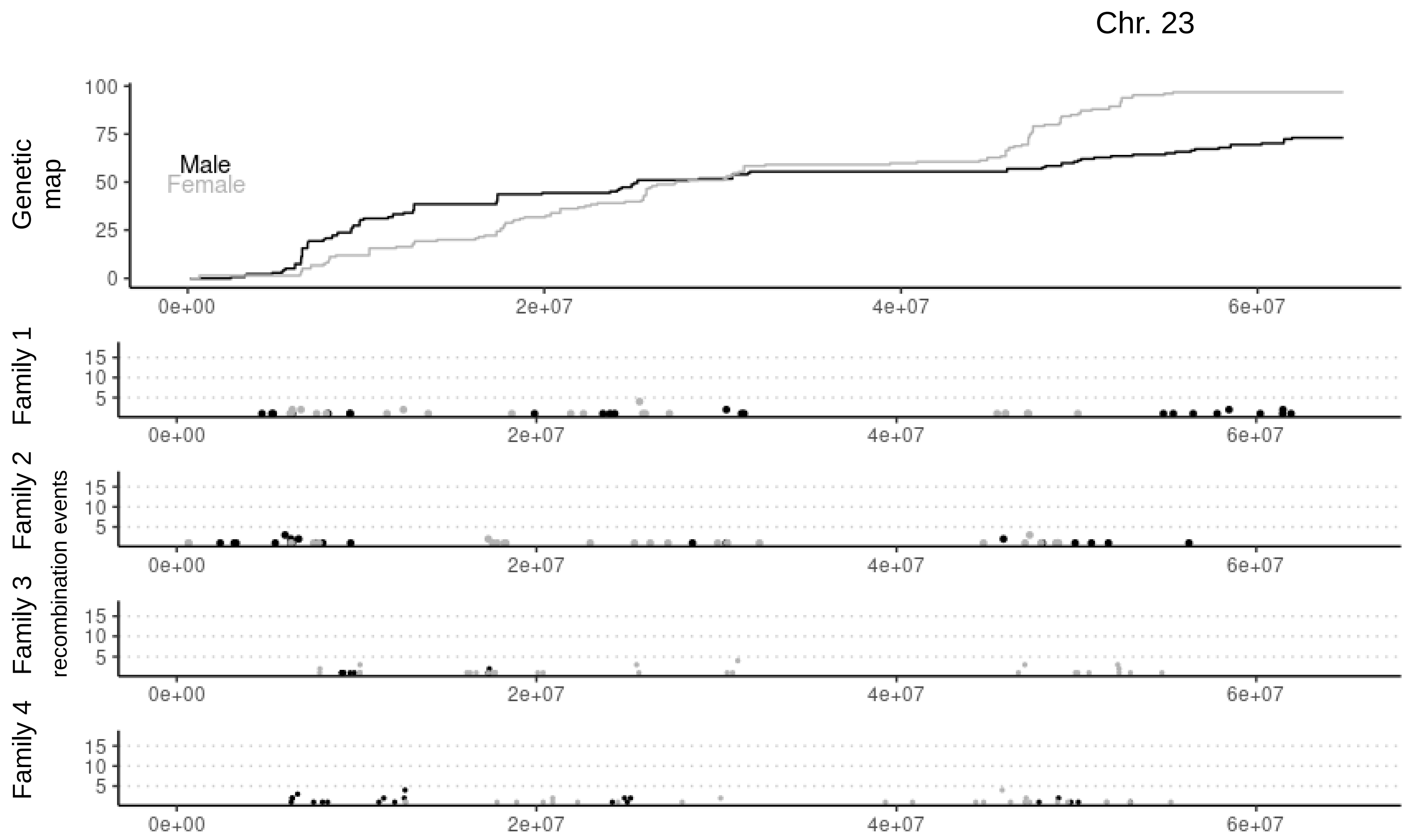

Chr. 23
Genetic map
Male
Female
100
75
50
25
0
0e+00
2e+07
4e+07
6e+07
Family 1
Family 2
Family 3
Family 4
recombination events
15
10
5

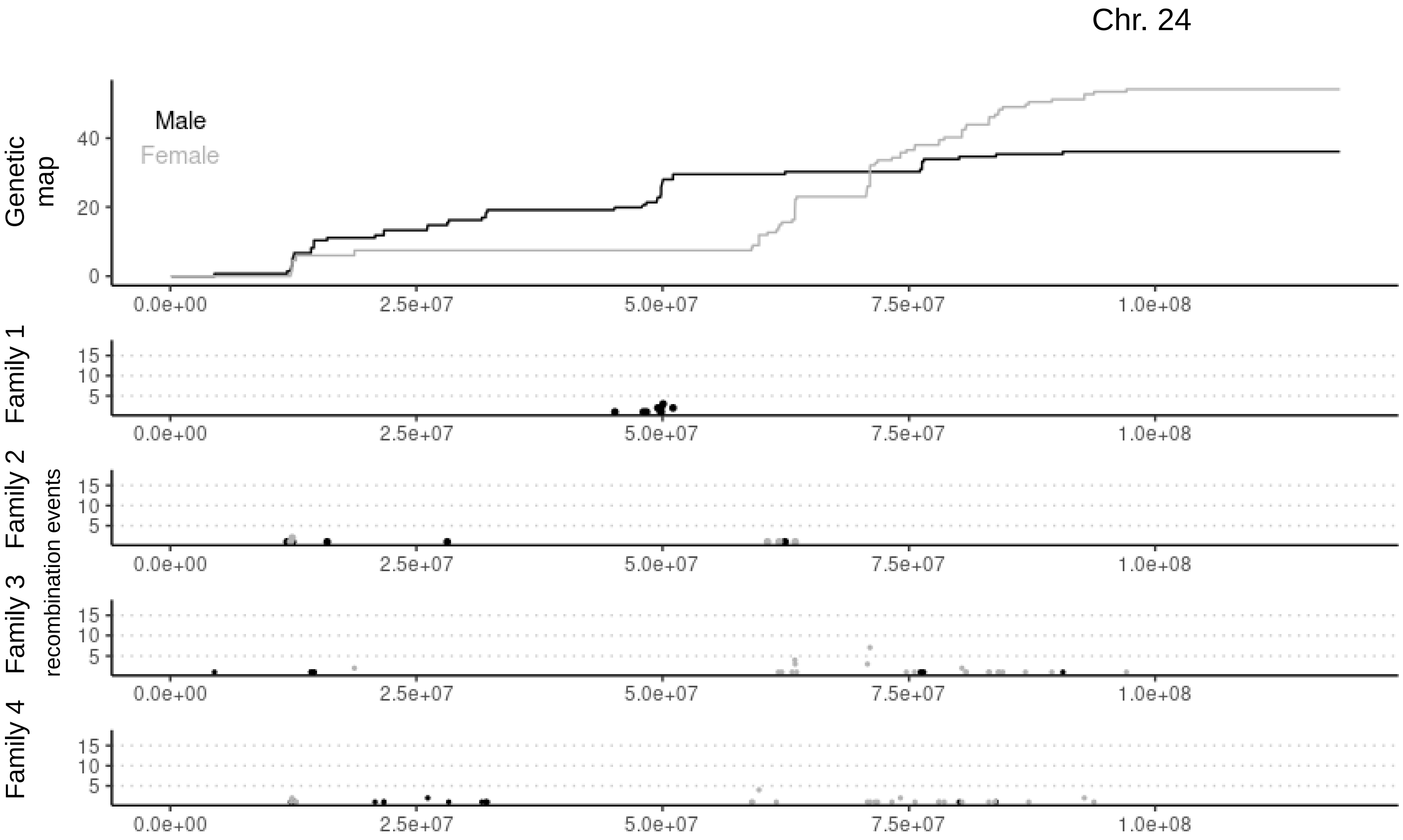

Chr. 24
Genetic map
Male
Female
40
20
0
0.0e+00
2.5e+07
5.0e+07
7.5e+07
1.0e+08
Family 1
Family 2
Family 3
Family 4
recombination events
15
10
5

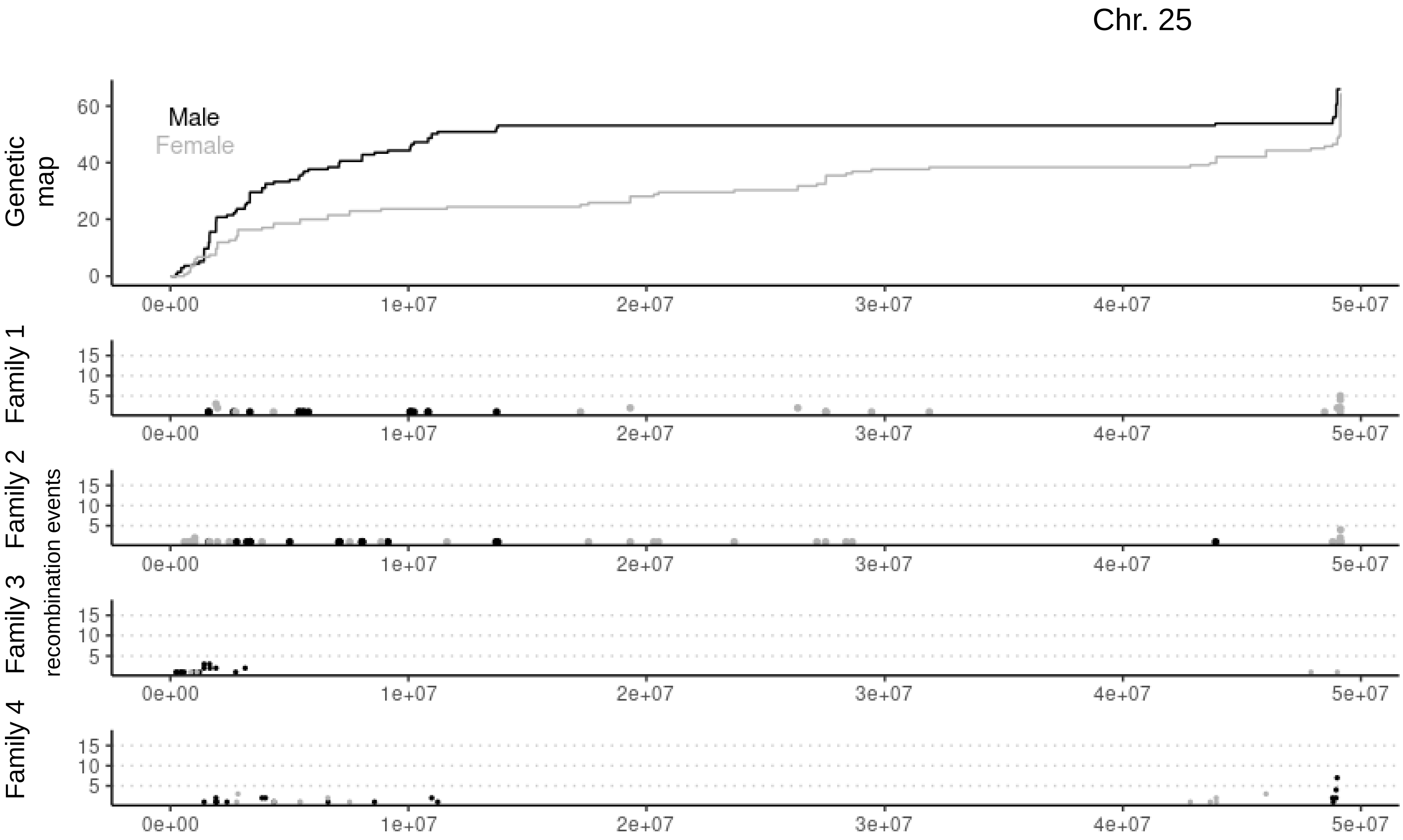
Chr. 25
Genetic map
Male
Female
recombination events
Family 1
Family 2
Family 3
Family 4
0
20
40
60
5
10
15
0e+00
1e+07
2e+07
3e+07
4e+07
5e+07

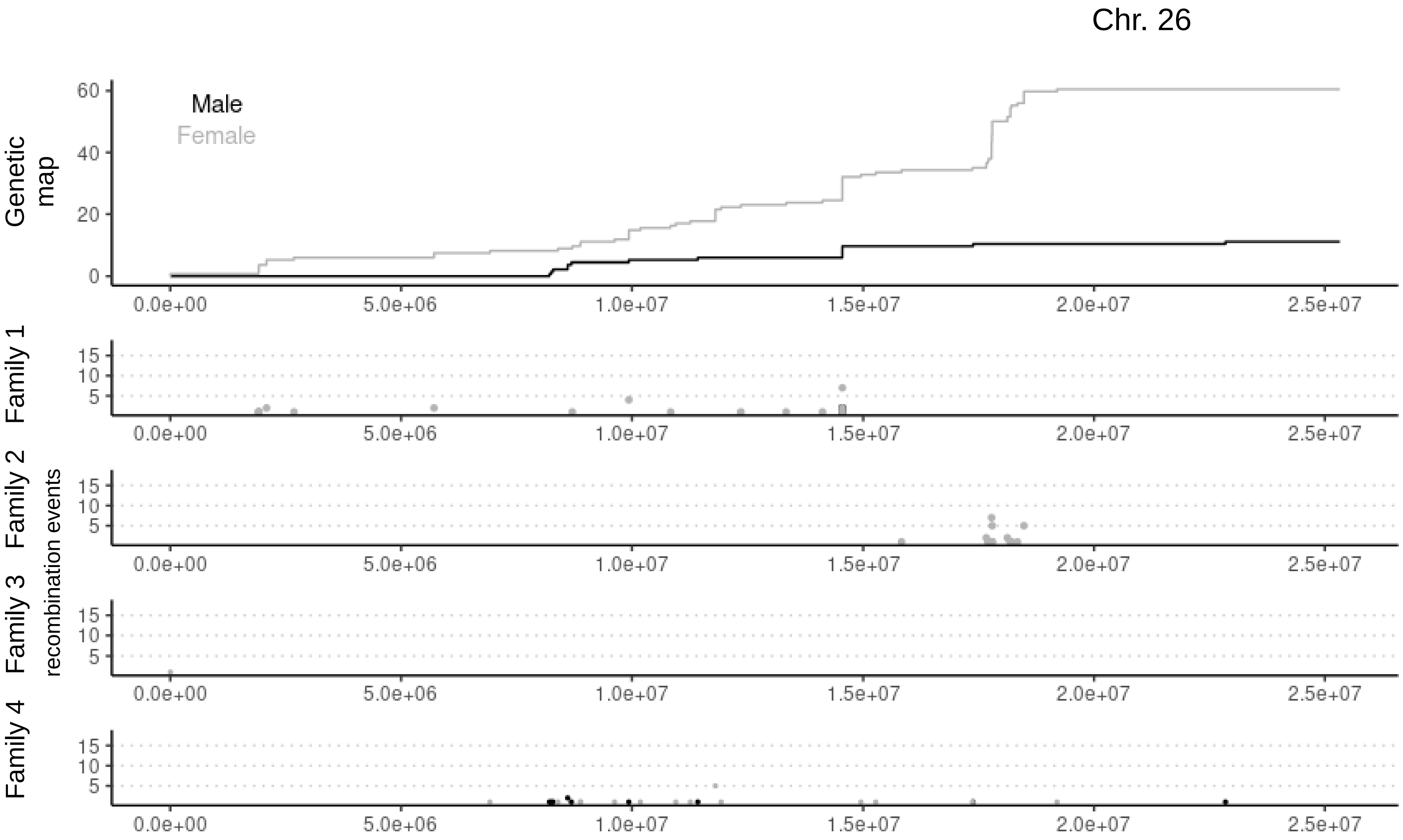
Chr. 26
Genetic map
Male
Female
60
40
20
0
0.0e+00
5.0e+06
1.0e+07
1.5e+07
2.0e+07
2.5e+07
Family 1
Family 2
Family 3
Family 4
recombination events
15
10
5

Chr. 27

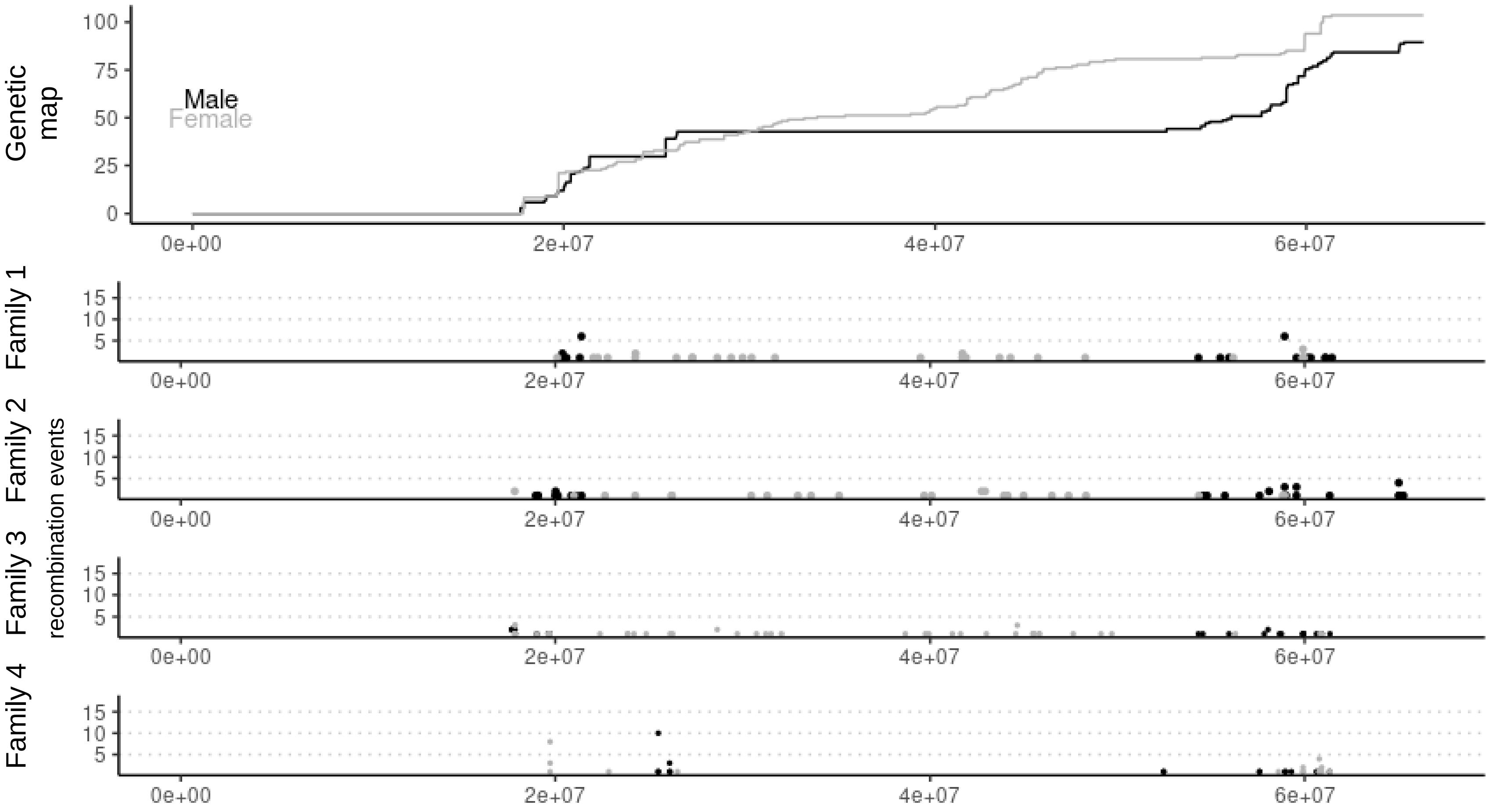

Genetic map
Male
Female
100
75
50
25
0
0e+00
2e+07
4e+07
6e+07
Family 1
Family 2
Family 3
Family 4
recombination events
15
10
5

Chr. 28

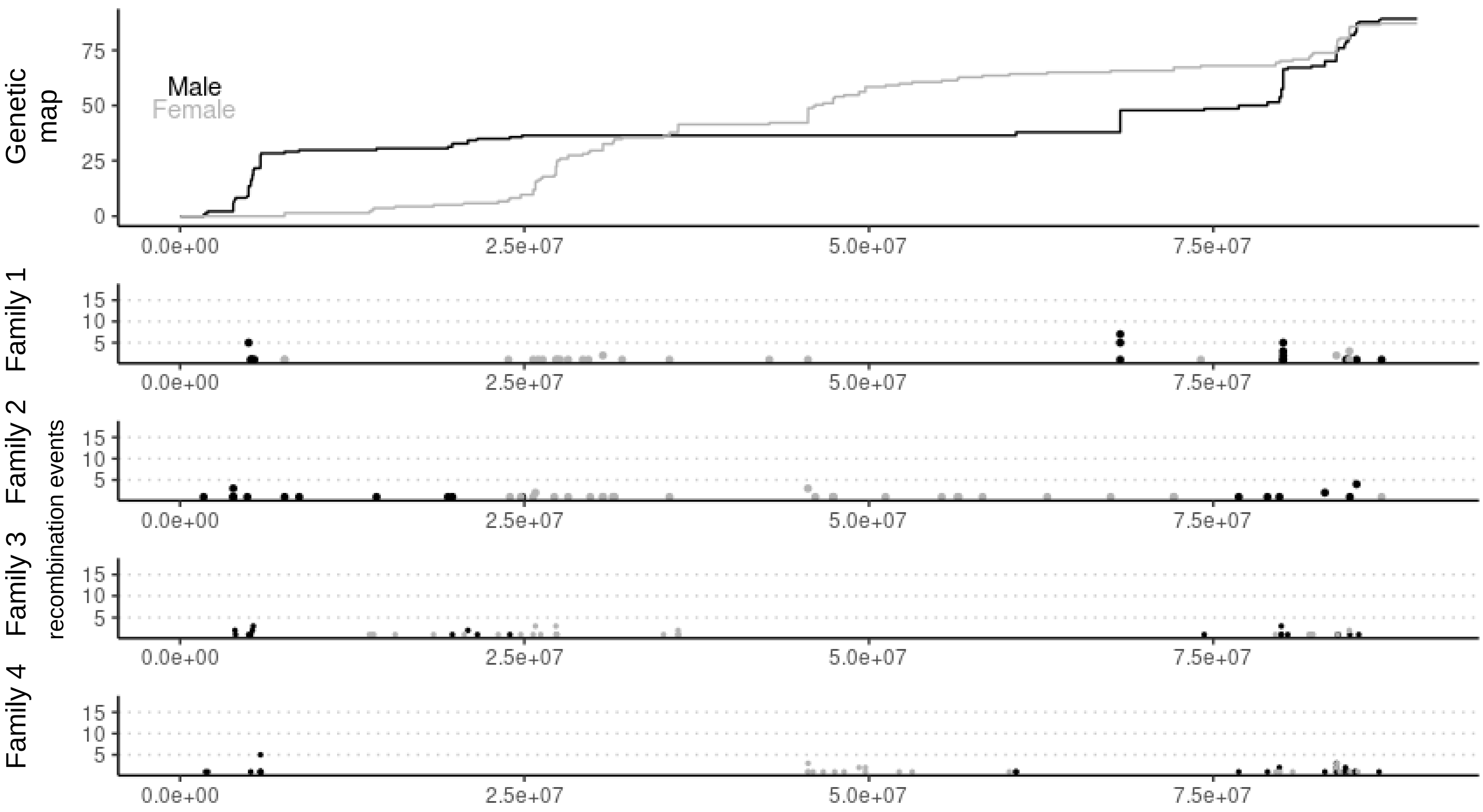

Genetic map
Male
Female
75
50
25
0
0.0e+00
2.5e+07
5.0e+07
7.5e+07
Family 1
Family 2
Family 3
Family 4
recombination events
15
10
5

Chr. 29

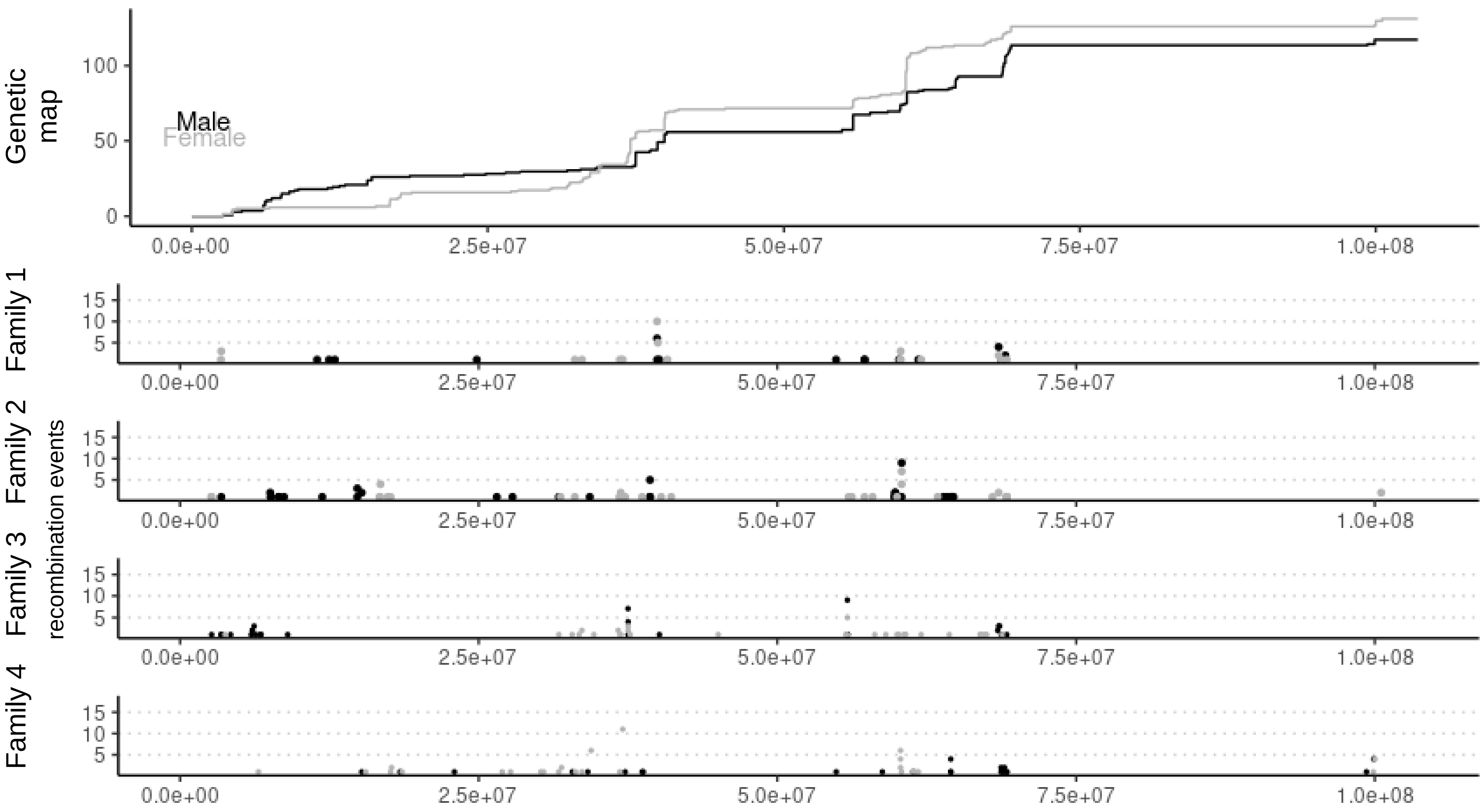

Genetic map
Male
Female
100
50
0
0.0e+00
2.5e+07
5.0e+07
7.5e+07
1.0e+08
Family 1
Family 2
Family 3
Family 4
recombination events
15
10
5

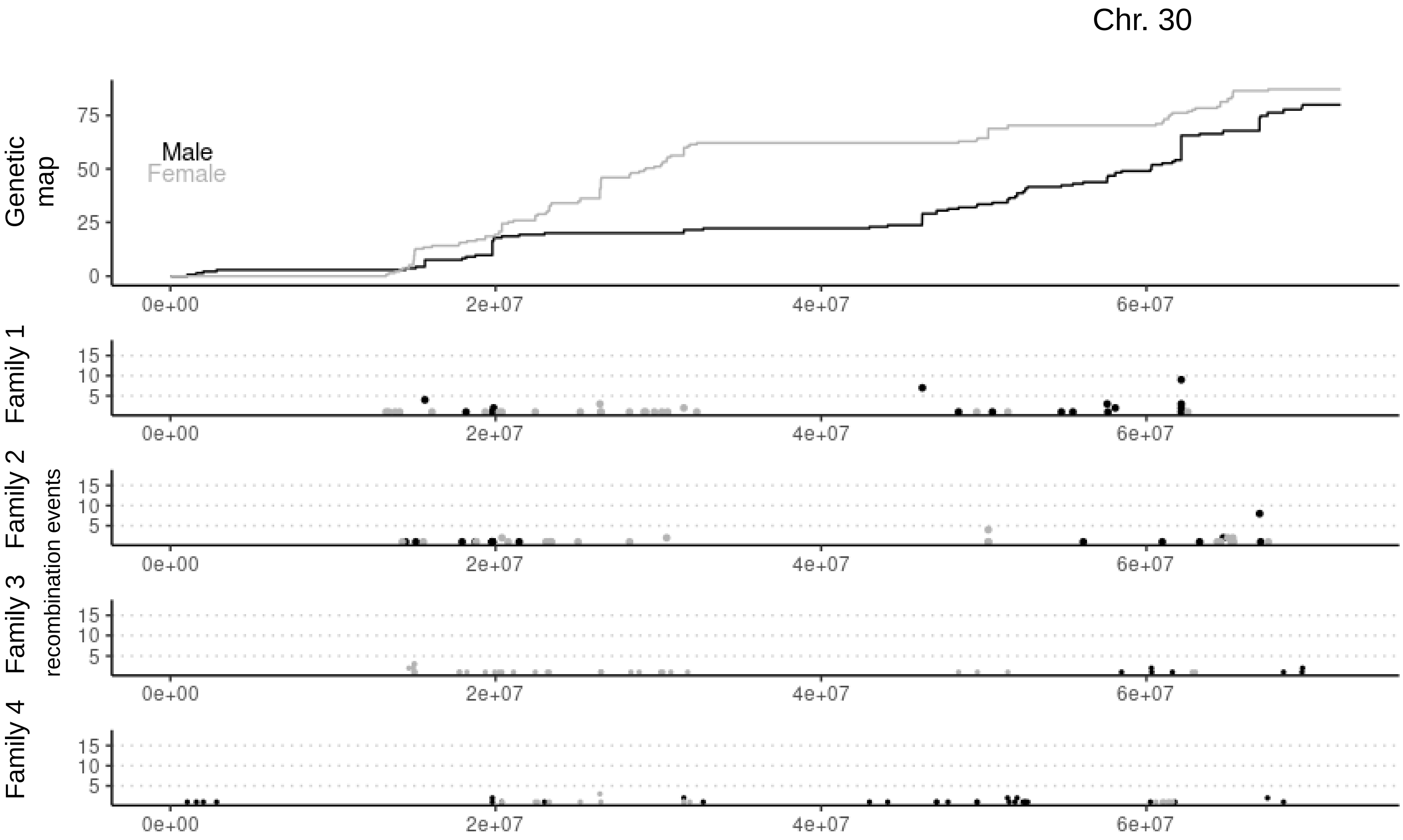

Chr. 30
Genetic map
Male
Female
0
25
50
75
0e+00
2e+07
4e+07
6e+07
Family 1
Family 2
Family 3
Family 4
recombination events
5
10
15

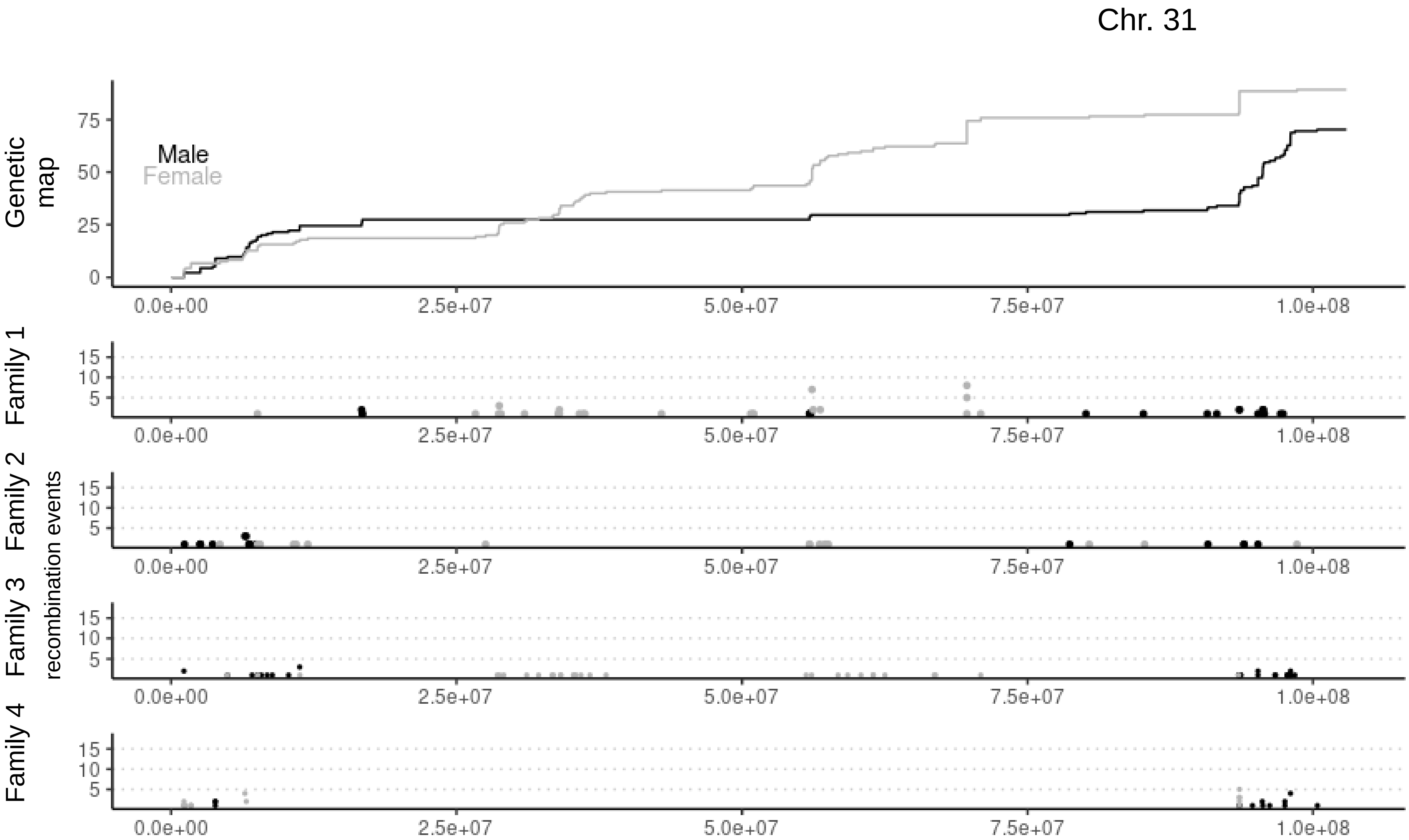
Chr. 31
Genetic map
Male
Female
75
50
25
0
0.0e+00
2.5e+07
5.0e+07
7.5e+07
1.0e+08
Family 1
Family 2
Family 3
Family 4
recombination events
15
10
5

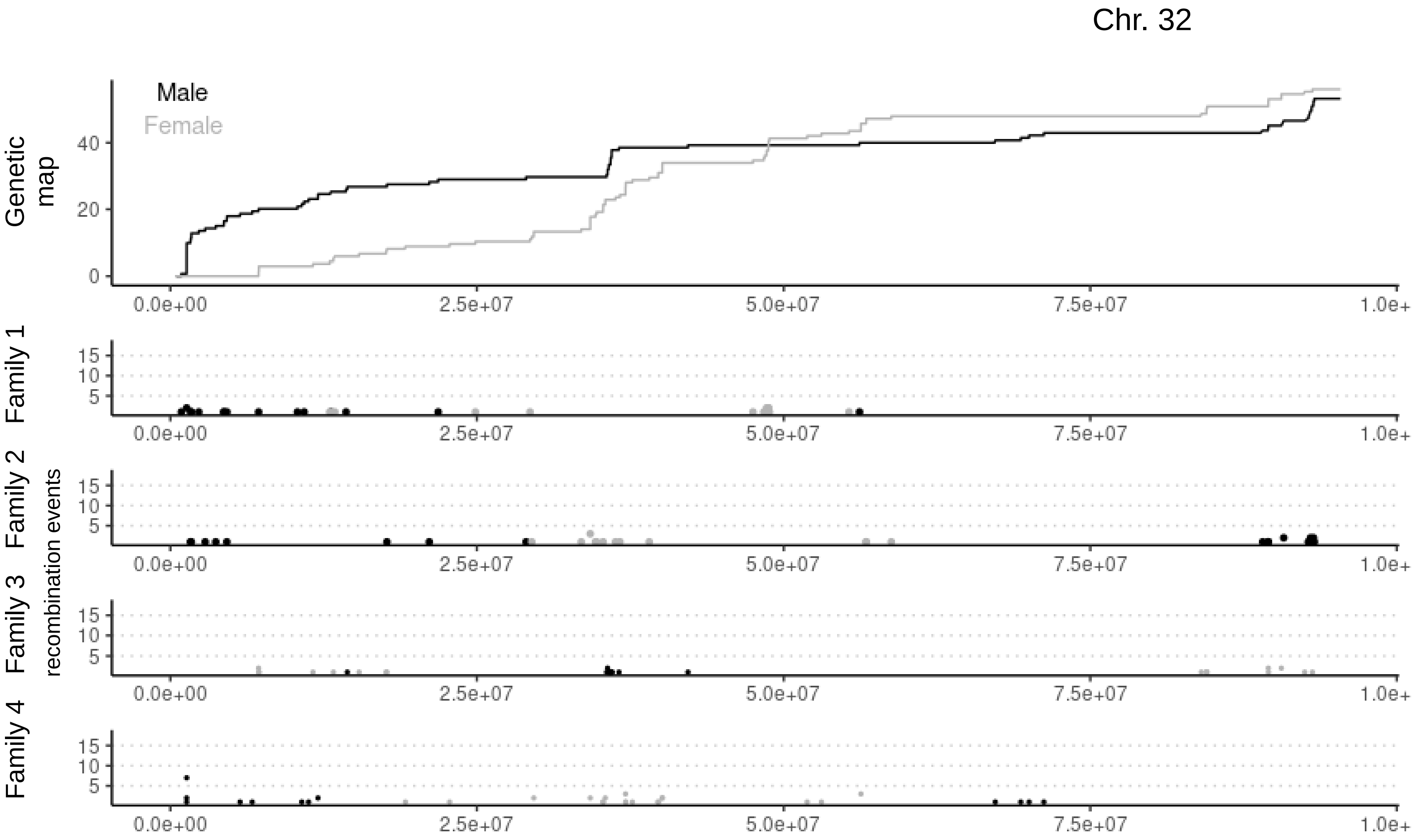
Chr. 32
Male
Female
Genetic map
0
20
40
0.0e+00
2.5e+07
5.0e+07
7.5e+07
1.0e+
Family 1
Family 2
Family 3
Family 4
recombination events
5
10
15

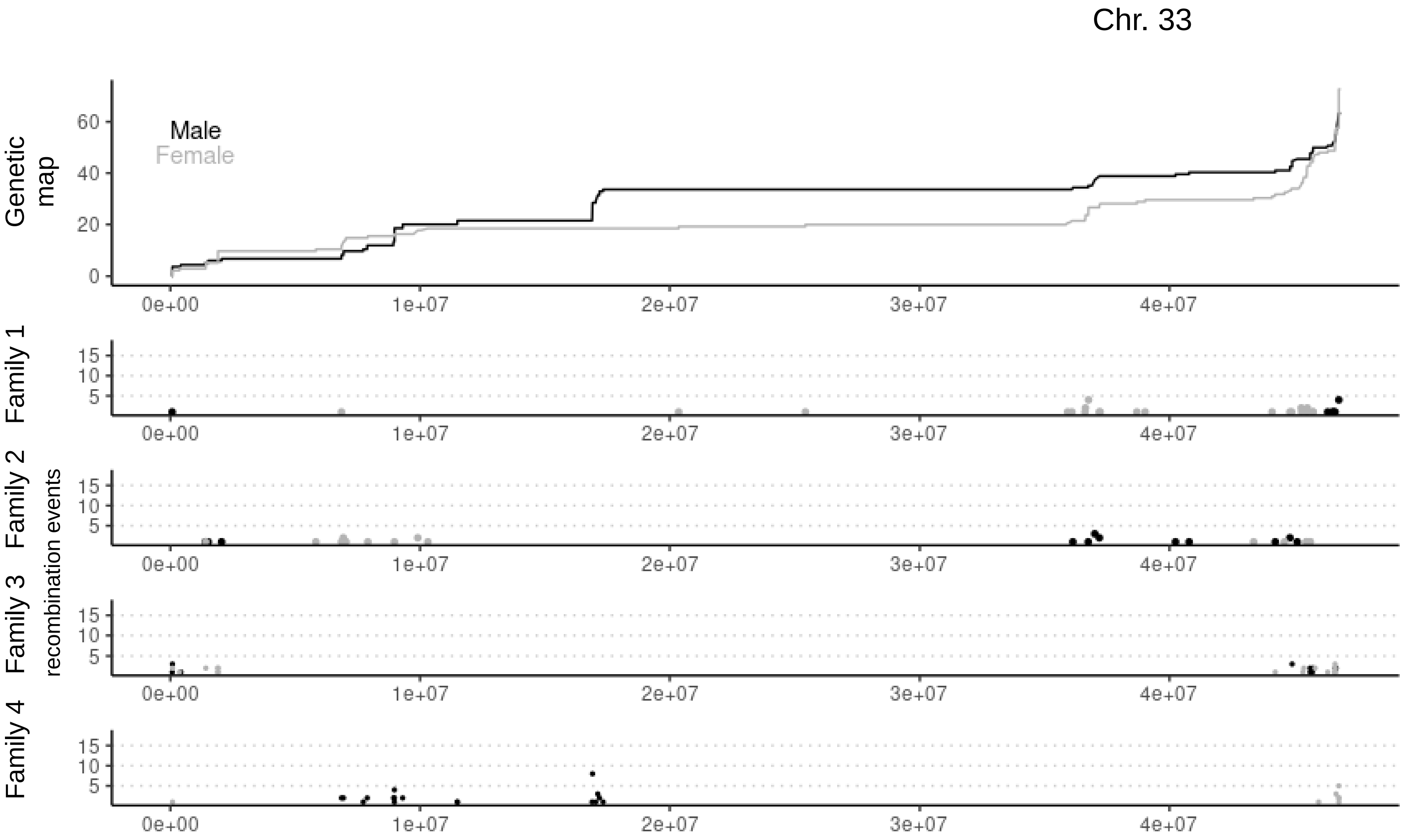

Chr. 33
Genetic map
Male
Female
60
40
20
0
0e+00
1e+07
2e+07
3e+07
4e+07
Family 1
Family 2
Family 3
Family 4
recombination events
15
10
5
