# Supplementary material for: Masu salmon species complex relationships and sex chromosomes revealed from analyses of the masu salmon (Oncorhynchus masou masou) genome assembly
Source: G3 (Bethesda). 2024 Nov 28;15(2):jkae278. doi: 10.1093/g3journal/jkae278 (PMC11797027; doi:10.1093/g3journal/jkae278)
Supplement: jkae278_Supplementary_Data [file jkae278_supplementary_data.zip › Supplemental_Legends_G3-2024-405504.docx]

**Supplemental Legends**

**Figure S1. Hi-C contact map.** a) Full Hi-C contact map, with chromosomes represented by the larger squares (blue) and contigs as smaller interior squares (green). A darker background (red) represents more dense contacts. Intra-chromosomal contacts have generally more dense contacts. b) Example of a homeologous region. In this example, the homeologous regions share a narrow diagonal strip of contacts even though these regions have strong support as independent chromosomes. A putative collapsed homeologous region has similar Hi-C contacts for both chromosomes. Notice the size of the contigs are smaller in this putative collapsed region. The smaller contig size and support for two locations made these regions more difficult to place contigs.

**Figure S2. Comparison of syntenic chromosomes in Pacific salmon - expanded.** In this comparison, all the reference genome assemblies for each species were aligned to the northern pike reference assembly. Each chromosome from each species is depicted and named based on its synteny with the northern pike. We used the designations of .1 (with box) and .2 to depict the first and second homeolog. This nomenclature and the assignments were taken from Sutherland *et al.* (2016). Each column represents the northern pike chromosome and the fusions that must have occurred to generate the chromosomes of the Pacific salmon. Fusions and fissions that were small were not shown for clarity. The locations of sex chromosomes are indicated with arrows. The sex chromosomes were determined from multiple studies in addition to the current (Phillips *et al.* 2005, 2006; Rexroad *et al.* 2008; Naish *et al.* 2013; Brieuc *et al.* 2014; Kodama *et al.* 2014; Palti *et al.* 2015; Larson *et al.* 2016; Sutherland *et al.* 2017; Pan *et al.* 2019; Gao *et al.* 2021; Christensen *et al.* 2021; Rondeau *et al.* 2023a). In masu salmon, the chr 25.2 and 15.1 fusion was inferred from alignments to other salmon species rather than a direct comparison to the northern pike. This is the same with the chr 23.1 and 24.1 fusion in coho salmon.

**Figure S3. Recombination events for each chromosome.** Same as Figure 4 (see legend), but for each chromosome.

**Table S1. Resequenced genome sample information.**

**File S1. Haplotig and artifact contigs.**
