# Supplementary figures and images for: Masu salmon species complex relationships and sex chromosomes revealed from analyses of the masu salmon (Oncorhynchus masou masou) genome assembly
Source: G3 (Bethesda). 2024 Nov 28;15(2):jkae278. doi: 10.1093/g3journal/jkae278 (PMC11797027; doi:10.1093/g3journal/jkae278)

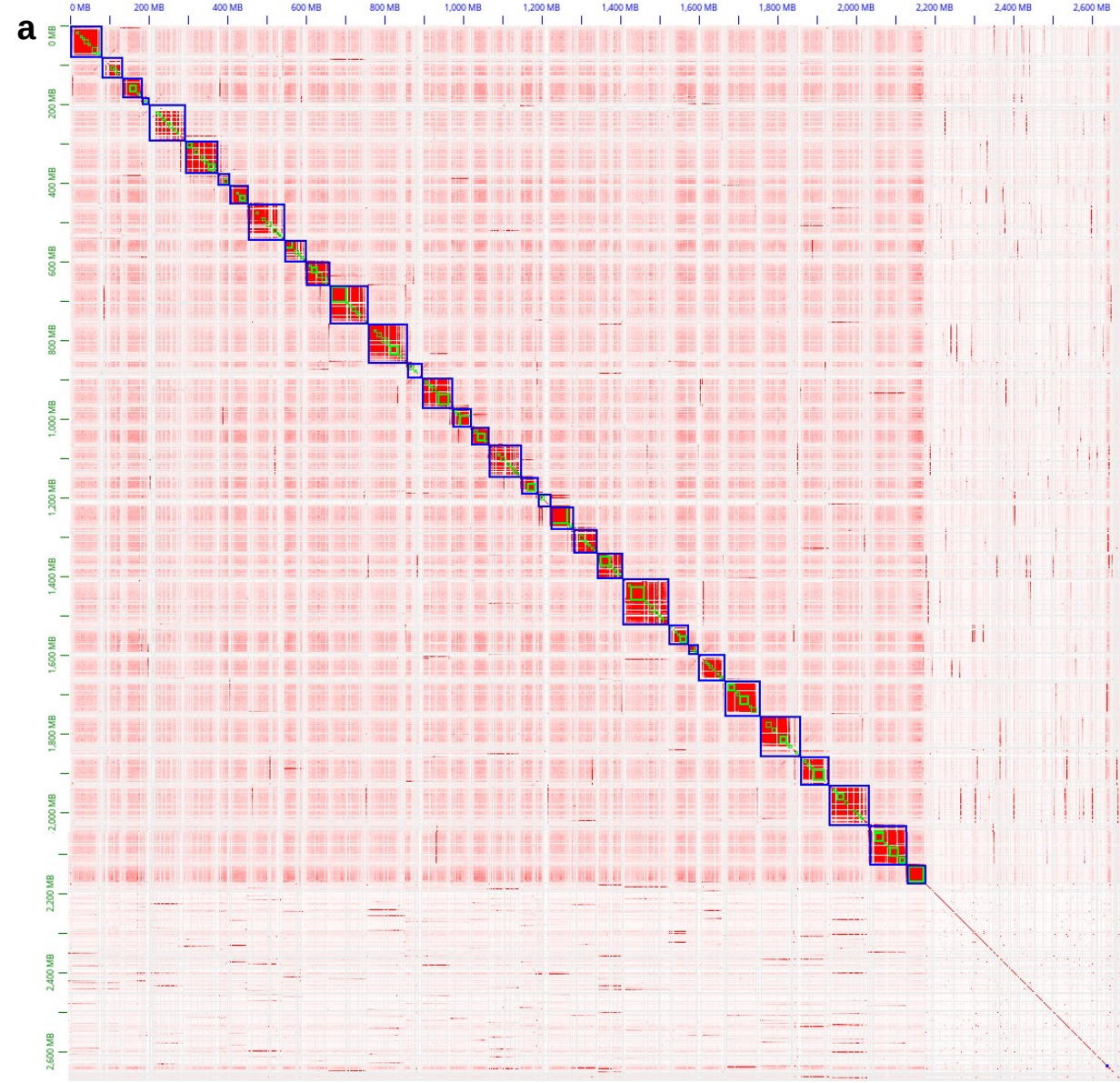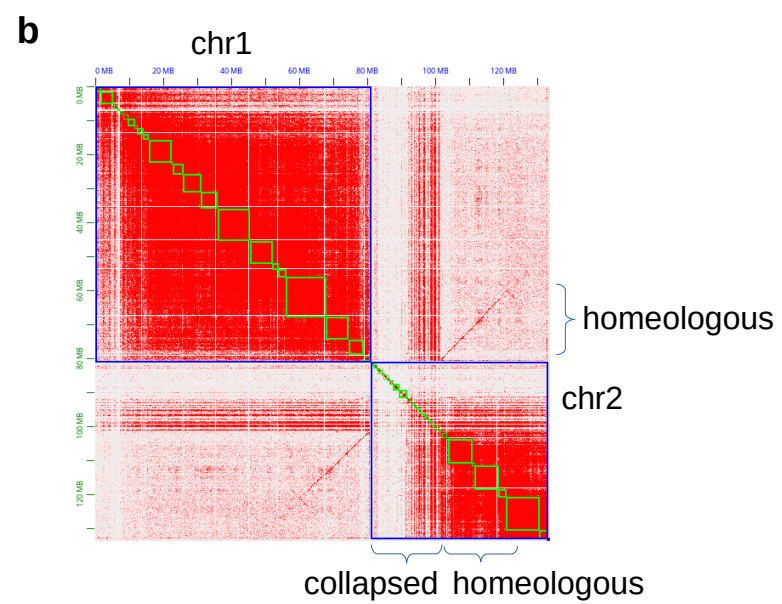

Supplement: jkae278_Supplementary_Data [file jkae278_supplementary_data.zip › Figure_S1_G3-2024-405504.pdf]

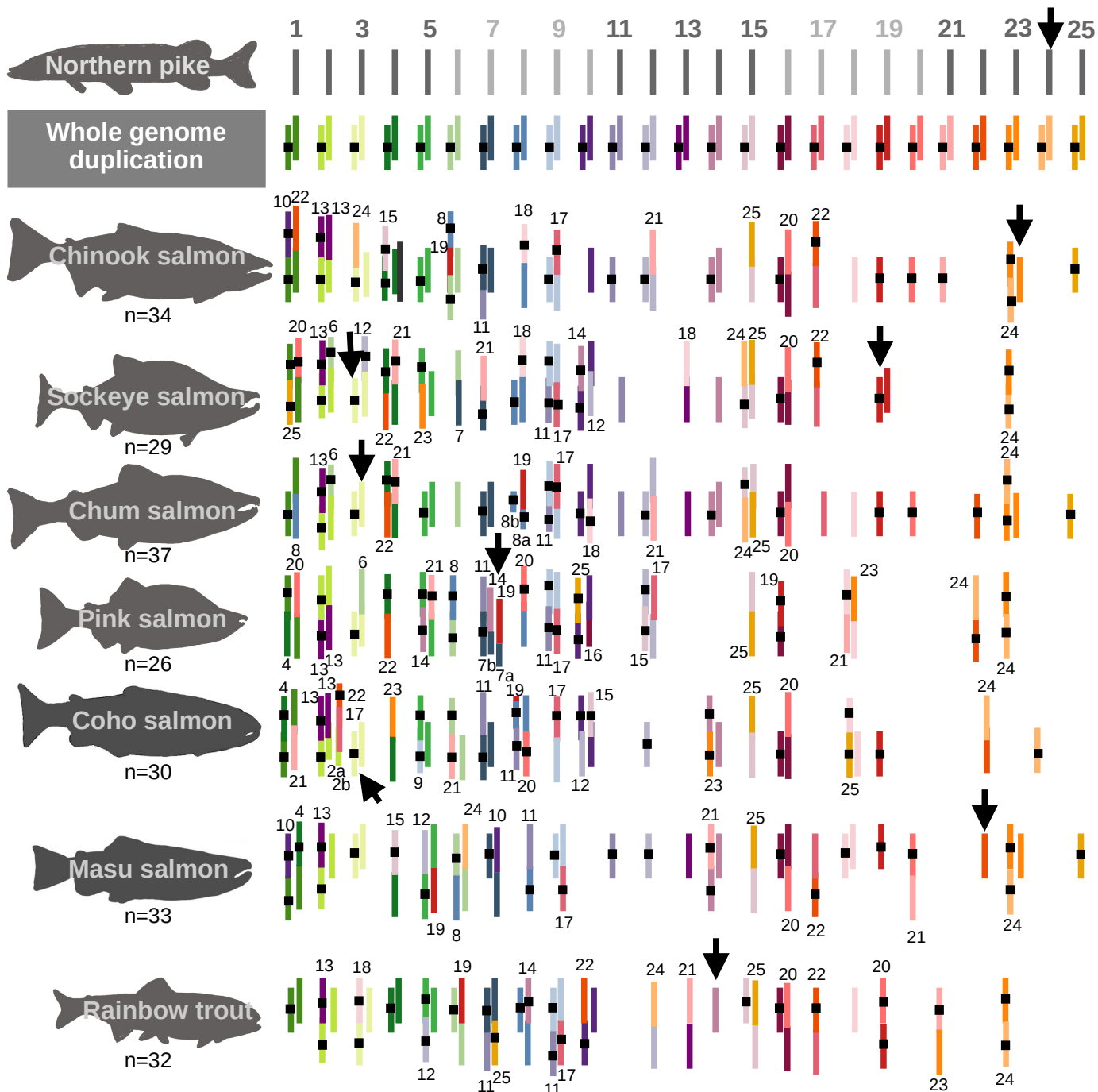

Supplement: jkae278_Supplementary_Data [file jkae278_supplementary_data.zip › Figure_S2_G3-2024-405504.pdf]
